# Imitating the winner leads to discrimination in spatial prisoner’s dilemma model

**DOI:** 10.1038/s41598-019-40583-w

**Published:** 2019-03-07

**Authors:** Gorm Gruner Jensen, Stefan Bornholdt

**Affiliations:** Institute for Theoretical Physics, University of Bremen, 28359 Bremen, Germany

## Abstract

The occurrence of discrimination is an important problem in the social and economical sciences. Much of the discrimination observed in empirical studies can be explained by the theory of in-group favouritism, which states that people tend to act more positively towards peers whose appearances are more similar to their own. Some studies, however, find hierarchical structures in inter-group relations, where members of low-status groups also favour the high-status group members. These observations cannot be understood in the light of in-group favouritism. Here we present an agent based model in which evolutionary dynamics can result in a hierarchical discrimination between two groups characterized by a meaningless, but observable binary label. We find that discriminating strategies end up dominating the system when the selection pressure is high, i.e. when agents have a much higher probability of imitating their neighbour with the highest payoff. These findings suggest that the puzzling persistence of hierarchical discrimination may result from the evolutionary dynamics of the social system itself, namely the social imitation dynamics. It also predicts that discrimination will occur more often in highly competitive societies.

## Introduction

Structural discrimination is a problem across a wide range of societies. Often, there is no apparent connection between the defining characters of a group and an obvious rational reason to discriminate against its members. One of the most commonly observed discriminating behaviours^1,2^, in-group favouritism, is characterized by a tendency to show more cooperation, preference, or altruistic behaviour towards people whose appearance is close to ones own (the in-group) than to those who appear different (the out-group). However, a growing body of research seems to indicate that members of some minority groups, particularly those of low social status, favour their in-group much less than members of high status groups, or even favour members of their high-status out-group (in-group devaluation)^3–11^. These relations can be thought of as a composition of a symmetric and an antisymmetric part. In-group favouritism can account for the symmetric part since a person, A, is in the in-group of another person, B, if and only if B is in the in-group of A. We will refer to the asymmetric part as hierarchical discrimination because it accounts for the phenomenon that members of one group has an overall advantage compared to members of the other. In-group favouritism clearly cannot explain why such hierarchies should be accepted by those they disadvantage. This has lead to the development of system justification theory, which states that people have a intrinsic tendency to legitimize and preserve systemic forms of inequality^12^. System justification theory provides an efficient explanation for the observed out-group favouritism from low-status group to high-status groups by introducing a fairly strong assumption about human psychology. This raises the question about whether this kind of behaviour could be explained from simpler, or more fundamental, principles.

Some investigations following the classical economic principle of selfish rationality have revealed an important connection between discrimination and incomplete information. A rational agent who has to choose between individual options belonging to different groups may try to compensate incomplete information about the quality of each choice by factoring in a prior knowledge about the quality distributions within each group. This could for example be an employer choosing between different employees, or a customer choosing between different suppliers. If the quality-distributions of the two groups are very different this knowledge is weighted heavily, it could lead the agent to choose an option with a weaker individual performance estimate, but belonging to an – on average – stronger group, instead of someone with a stronger individual performance estimate, but belonging to a weaker group^13^. This statistical theory of discrimination has been used as a fundamental building block for designing dynamical models in which the collective reputation of group will be trapped in one of a number of possible Nash-equilibria stabilized by positive-feedback between the expected and the optimal behaviour^14,15^. These types of models suggest that persistent discrimination can be explained without assuming any differences of intrinsic properties of the members of different groups. All the existing models of this kind, however, describe asymmetric interactions, where the ‘discriminating’ agent belongs to a completely different category than the ‘discriminated’ agents. Thus, none of them directly describes in-group devaluation. Also, there may not be a simple way of introducing it in this framework, because a rational agent would be expected to recognize that in-group devaluation would negatively affect its own future possibilities, and avoid acting against its own self-interest. One way to circumvent this problem could be to loosen the assumption of rationality. This could be further motivated if the discriminating behaviour often occurs unconsciously, or indirectly via complex social interactions which are not fully understood by the agents.

Evolutionary game theory is one framework which has been enormously effective in explaining social phenomena such as altruism and cooperation^16^. In contrast to the classical economical theories it approaches the behavioural question starting with almost completely irrational agents who do not have any direct knowledge about the consequences of their actions. Rather than making assumptions about the actual decision process, it works by assuming that behaviours are fundamentally random, but successful strategies are promoted by replicating at a higher rate. This replication is commonly interpreted either as biological reproduction of genes or, in the context of cultural evolution, as mimicking of ideas or behavioural motives. A number of studies have investigated how the introduction of more or less arbitrary tags can be used to promote the evolution of cooperation^17–22^. More recently, a study has investigated the effect of tags when considered in combination with population structure — another mechanism well known to promote cooperation^23^. There it was shown that the introduction of tags could also reduce the overall level of cooperation, by allowing discriminating strategies to invade a population of unconditional cooperators. It has been pointed out by Fu *et al*.^20^ that this mechanism can be used to explain the evolution of in-group favouritism within dynamic groups where memberships may change quickly, e.g. political movements. The existing models of tag-based cooperation, however, do not reproduce the observed phenomenon of sustained hierarchical discrimination, i.e. a mixed population in which members of one group are treated preferentially, both by their peers, as well as by members of the less fortunate groups.

Here we suggest a new model for investigating hierarchical discrimination in the framework of evolutionary game theory. Starting from an established model describing the evolution of cooperation in a prisoner’s dilemma type game on graphs^24^, we partition the population into two groups by randomly assigning an observable, but completely meaningless, binary label to each agent (blue or green). As the labels are observable, this expands the set of possible strategies from two (*cooperate* and *defect*), to four (1: *cooperate with all*, 2: *cooperate only with blue*, 3: *cooperate only with green*, 4: *defect all*). We call strategies discriminating when they imply different behaviour towards agents carrying different labels. An agent can exhibit in-group favouritism if its strategy is only to cooperate with neighbours carrying the same label as itself, and in-group devalueating if it only cooperates with neighbours carrying the other label. We say that the system is displaying hierarchical discrimination if a large group of agents with mixed labels all agree only to cooperate with those carrying one of the labels.

In contrast to models of tag-based cooperation, the labels described in this model never change. Hence the only dynamical variable in the model are the strategies, which agents change mainly by imitating their neighbours. As strategies spread from agent to agent, the model has a tendency to let one strategy dominate all (or at least large regions) of the population. Thus our main task is to investigate which conditions (choices of parameters) lead to the dominance of discriminating strategies.

## Model

Let us define a game of agents distributed on a graph. Each agent has a static and binary ‘label’, either green or blue, decided at the beginning of the game. This label serves as the only observable difference between agents. Agents interact with their nearest neighbours in a prisoner’s dilemma type of game where they can either cooperate or defect. When cooperating, an agent donates one unit of value to the neighbour. To simulate the benefit of cooperation, the donation is scaled by a constant factor *b* > 1, such that the neighbour receives *b* times the unit value. When an agent is defecting, no value is transferred. Since agents cannot distinguish between neighbours who have the same label they must act the same way towards all of them. Thus the model has four possible strategies: “defect all”, “cooperate with all”, “cooperate only with green”, and “cooperate only with blue”.

**Figure 1:**
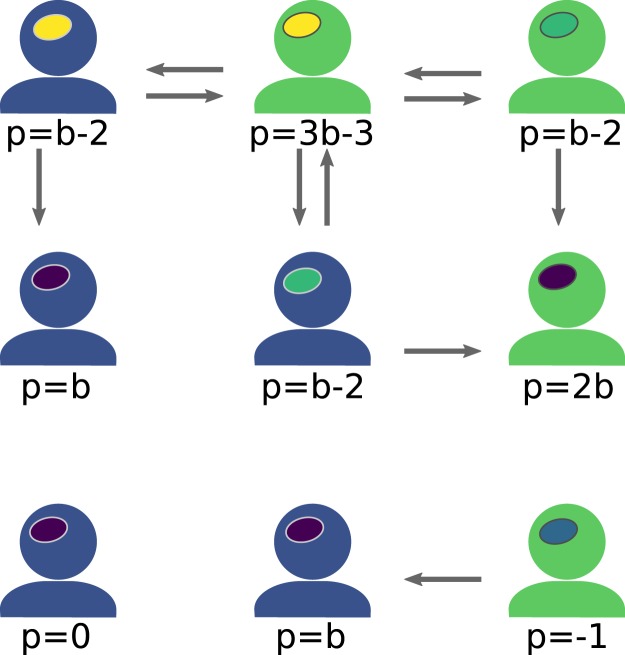
Model description: Agents are located on a square lattice. Each agent has a label (blue or green) which can be observed by their neighbours, and a strategy (yellow: “cooperate with all”, green: “cooperate with green”, blue: “cooperate with blue”, purple: “defect all”) which determines their interaction with their neighbours. From this configuration, an agent’s payoff *p* is calculated by subtracting 1 for each neighbour it cooperates with, and adding *b* for each neighbour cooperating with it. Here the parameter *b* represents the benefit of cooperation. Each time-step one randomly chosen agent changes its strategy by copying that of one of its neighbours. This neighbour is chosen with a probability proportional to *f* = exp(*wp*), where the parameter *w* represents the selection pressure. When the selection pressure is small ($w\to 0$) neighbours are chosen with almost equal probability independent of their payoff. When the selection pressure is high ($w\to \infty$) the neighbour with the highest payoff will almost certainly be chosen.

Given a configuration of labels and strategies, a payoff, *p*, can be calculated for each agent. An agent’s payoff is the sum of all donations the agent is receiving from its neighbours, minus all the donations it is giving away. Notice that the payoff of an agent *i* does not accumulate over time, and can always be calculated from the current state by:

$${p}_{i}=\sum _{j\in {{\mathscr{N}}}_{i}}\,b\cdot {S}_{j}({\lambda }_{i})-{S}_{i}({\lambda }_{j}),$$

where ${{\mathscr{N}}}_{i}$ is the set of neighbours of agent *i*, ${\lambda }_{i}\in \{{\rm{blue}},{\rm{green}}\}$ is the label of agent *i*, and ${S}_{i}({\lambda }_{j})=1$ if the strategy of agent *i* is to cooperate with the label worn by agent *j*, and ${S}_{i}({\lambda }_{j})=0$ if it is to defect. Figure 1 illustrates a simple example of how the calculation work.

The evolutionary dynamics of the spreading of the strategies is as follows. Every turn a random agent is chosen (from a uniform distribution over all agents). With a small probability *μ*, this agent will mutate, i.e. choose a new random strategy (from a uniform distribution over all strategies). Else, with probability 1 − *μ*, the chosen agent will imitate the behaviour of one of its neighbours. In that case, a neighbour is chosen with a probability proportional to its fitness *f* which is directly related to its payoff by:

$${f}_{i}={e}^{w\cdot {p}_{i}}$$

where *f*_*i*_ and *p*_*i*_ are the fitness and payoff of agent *i*, and *w* is a model parameter controlling the selection pressure. When the selection pressure approaches zero, all neighbours are chosen with almost equal probability. When the selection pressure is high, the neighbour with the highest payoff will almost certainly be chosen.

Our model is characterized by three parameters: The cooperation benefit *b*, the selection pressure *w*, and the mutation rate *μ*.

The random mutations serve two functions: Adding noise and preventing strategies from going extinct. We are interested in the evolutionary dynamics of imitation, and not in the noise. Thus we keep the mutation-rate small and constant (*μ* = 0.001) throughout this paper.

In addition to the three parameters mentioned above we also have to specify both the topology of the underlying graph and the distribution of labels, in order to have a well-defined dynamical system. In this paper we have chosen to focus on a 2-dimensional square lattice, because it allows for intuitive visualization of the model-state. A natural and easy choice for the label-distribution is to randomly assign the label of each agent independently. In this paper we highlight a variation of this, in which the probability that an agent is given a green label increases linearly from zero in one end of the lattice to one in the other end. We emphasize this distribution because it allows us to clearly demonstrate the important effect of having a regional majority. When studying agent-based models on lattices, it is customary to use periodic boundary conditions to avoid boundary effects and maximize symmetry. However, since we have already broken the symmetry in one direction by introducing a gradient in the label density, it seems unnatural to connect the two ends which have “opposite” population compositions. Our lattice, therefore, only has periodic boundary conditions in the direction perpendicular to the label distribution gradient, thus forming a cylinder.

It is beyond the scope of this paper to make a detailed discussion about the effects of using different graph-topologies or label distributions. We do, however, refer interested readers to the supplement for results with uniform label-distribution on a 2D lattice, Erdős-Rényi random graphs, and a 1D ring-topology (Supplement Section 1).

The model described here is an extension of a model introduced by Ohtsuki *et al*. in an effort to study how spatial structures may promote evolution of cooperation^24^. The most important change is the introduction of the observable labels. However, we also differ by defining the fitness using the exponential function ${f}_{i}={e}^{w\cdot {p}_{i}}$, whereas they choose $f=1-w+w\cdot p$. In the limit of low selection pressure ($w\to 0$), these two definitions converge. The choice of the exponential function, however, secures that all fitnesses will be positive no matter how high the selection pressure. This allows us to investigate all the way to the deterministic limit, where agents always imitate the neighbour with the highest payoff.

## Results

**Figure 2:**
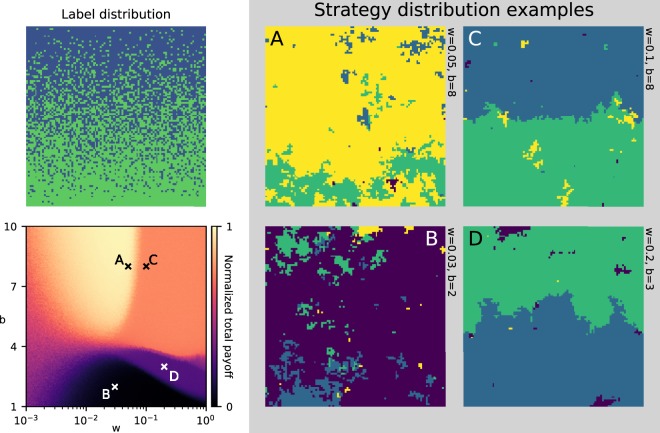
Phase-diagram. Constant mutation rate *μ* = 0.001 and grid-size 100 × 100 agents. Top-left: The label distribution used in each of the examples to the right. In the parameter scan, a new label distribution is generated at every point, to ensure that the results are not unexpectedly caused by random local structures. Bottom-left: Parameter scan over cooperation benefit *b*, and selection pressure *w*. The colour indicates the mean payoff normalized, averaged over 20 samples uniformly distributed over a period of 10^8^ time steps. At each data point the system is initialized with all defectors and run for a transient period of 2 × 10^7^ time steps before the mean payoff is measured. Right: Four snapshots of strategy-distributions at parameters corresponding to those marked in the parameter scan. The four strategies are colour coded as follows; yellow: “cooperate with all”, green: “cooperate with green”, blue: “cooperate with blue”, purple: “defect all”. The snapshots are taken after 4 × 10^7^ timesteps.

Figure 2 captures the long-term behaviour of the model, as it settles into different stationary states dependent on parameters.

In the bottom left panel the normalized mean payoff is plotted (in colour) as a function of the selection pressure *w* and the cooperation benefit *b*. The normalized mean payoff is identical to the fraction of donations in the system out of all possible ones. Due to the stochastic nature of the model, the system is never locked in an absorbing fixed point. The normalized mean payoff, however, tends to stabilize after some transient period, as the system finds a stationary distribution of strategies. To capture this long term behaviour, we initialized the system with all defectors and run the model for a transient period of 2 × 10^7^ time steps before the mean payoff is measured. We then measure the averaged value over 20 samples evenly spaced over a period of 10^8^ time steps. The phase diagram clearly shows four distinct regions characterized by different levels of cooperation.

In the right panel, four examples of stationary state strategy distributions are shown, one for each phase. These snapshots show the following characteristics, which we have observed consistently throughout all our simulations:

**A**: The system is dominated by full cooperation resulting in close to maximal mean payoff. As expected, this optimal state requires that the cooperation benefit is greater than 4 (average connectivity), as predicted by Ohtsuki *et al*.’s simple rule for the evolution of cooperation on graphs and social networks^24^. Surprisingly, we find that it is also necessary to have a relatively low selection pressure to obtain this state of almost full cooperation.

**B**: When the cooperation benefit is low, the dominant strategy is complete defection, resulting in approximately zero payoff. Surprisingly we find that when the selection pressure is high ($w\gtrsim 0.03$), full defection is outperformed by strategies with non-zero cooperation even when the cooperation benefit is less than four (the average connectivity).

**C**: In general, when the selection pressure is high, we observe that the system is dominated by the asymmetric, or discriminating, strategies. The result is the formation of regional hierarchies, where all agents cooperate with neighbours carrying one of the labels, independent of their own label. Thus the ‘upper-class’ benefit from the cooperation from all of their neighbours while only donating to their in-group. The exploited agents, on the other hand, display in-group devaluation by only cooperating with members of their out-group.

These hierarchical regions have an interesting connection with the local label density. With our choice of label distribution, the lattice of agents is split into two qualitatively different regions. In ‘the top’ of the cylinder, the majority of agents have the blue label, while in ‘the bottom’ the green label is much more common. When the cooperation benefit is high, $b\gtrsim 4$ (average connectivity), we find a threshold for the selection pressure, above which the dominating strategy within each of the two domains (‘top’ and ‘bottom’) is to cooperate with the local majority label, while defecting against those in the minority. As a result, the normalized mean payoff is approximately equal to the population fraction of the majority in each domain, i.e. 3/4 given this specific label-distribution.

**D**: For high selection pressures and intermediate selection benefits *b* slightly below 4 (the average connectivity), we observe that domains of the asymmetric cooperation strategies have switched around as compared to the case with larger cooperation benefit *b* > 4 (the average connectivity). Cooperating with agents carrying one label, and defecting those carrying another, still out-competes both of the symmetric strategies, but within each of the two domains (‘top’ and ‘bottom’) the agents in the majority are defected, and only those in the minority group receive donations. Consequently, the normalized mean payoff is equal to the fraction of minority labels within each domain which is 1/4 with the given label-distribution.

Closer examination of the parameter scan in Fig. 2 reveals that the transition between the different phases are qualitatively different. For low selection pressure (left side of the figure), there is a smooth transition between ‘low mean payoff’ when the cooperation benefit is low, to ‘high mean payoff’ when the cooperation benefit is high. For intermediate cooperation benefits, we find very noisy stationary distributions in which patches of all possible strategies coexist. These high-entropy distributions are easy to understand. Because of the very low selection pressure the boundaries between strategy patches perform a random walk almost without a drift. When *w* = 0 dynamics are equivalent to a classical voter-model^25,26^ with four opinions.

The sharp phase transitions observed at higher selection pressures ($w\gtrsim 0.03$) are more intriguing because 1: The sharp transitions indicate that relatively small changes in the environment can have dramatic effects on the social structure, and 2: The sharp transitions separate the regions dominated by discriminating strategies.

The most intriguing finding in this new model, is the symmetry breaking phase transition in which full cooperation is ousted by a strategy of selectively cooperating with agents carrying one of the labels but not with those carrying the other label. Surprisingly, we find that, as long as the cooperation benefit is high (somewhat higher than the average connectivity), transition-point is determined almost solely by the selection pressure. In fact it seems that for selection pressures above a certain threshold, full cooperation cannot be restored no matter how much the cooperation benefit is increased. To get a better understanding of this, we have performed a detailed study of a simplified scenario.

In this example, all agents are arranged on a one-dimensional line rather than a square lattice. Each agent has only two neighbours. The mutation-rate is set to zero, so to avoid absorbing states we fix the strategy of the outermost agent in one end of the line to always being “full cooperation”, and the strategy of the outermost agent in the other end to “cooperating with blue, but defecting green”. Any strategy squeezed in between these two will eventually disappear (by random fluctuation), and the complete state of the system can be described by one number indicating the location of the boundary between one strategy and the other. The label distribution which we have studied in closest detail is one in which all agents have the blue label except for one “green” agent placed in the middle of the line. Notice that outside the neighbourhood of the one green agent, these two strategies lead to identical behaviour, and thus the boundary between them will simply make an unbiased random walk. Thus we believe this label distribution to be a good approximation for a more general scenario consisting of a large majority of blue labels, and a few green labels scattered over the line. Within 3 steps of the green agent, however, the boundary will move left or right with a probability that must be calculated explicitly for each position. After having determined the individual stepping probabilities we calculate the probability of finding it on the symmetric side of the green agent (the asymmetric strategy dominates) and the probability of finding it on the asymmetric side (the symmetric strategy dominates), assuming a stationary distribution:

$$\frac{p({\rm{d}}{\rm{i}}{\rm{s}}{\rm{c}}{\rm{r}}{\rm{i}}{\rm{m}}{\rm{i}}{\rm{n}}{\rm{a}}{\rm{t}}{\rm{i}}{\rm{o}}{\rm{n}}\,{\rm{d}}{\rm{o}}{\rm{m}}{\rm{i}}{\rm{n}}{\rm{a}}{\rm{t}}{\rm{e}}{\rm{s}})}{p({\rm{f}}{\rm{u}}{\rm{l}}{\rm{l}}\,{\rm{c}}{\rm{o}}{\rm{o}}{\rm{p}}{\rm{e}}{\rm{r}}{\rm{a}}{\rm{t}}{\rm{i}}{\rm{o}}{\rm{n}}\,{\rm{d}}{\rm{o}}{\rm{m}}{\rm{i}}{\rm{n}}{\rm{a}}{\rm{t}}{\rm{e}}{\rm{s}})}=\frac{{e}^{2w}}{\tanh \,(wb)+1}.$$

When the fraction is greater than one, we expect a strategy of asymmetric cooperation to be able to outperform the full cooperation in a mixed population. Comparison with the phase diagram for the one-dimensional system show a close, but non-perfect match (see Supplement Section 1.3). In the limit of very high cooperation-benefit, $b\gg 1$, the condition reduces to $w > \,\mathrm{log}\,(2)/2$. More details about the calculations can be found in the supplementary material. This result is consistent with the visual impression from the parameter-scan in Fig. 2, that for high enough selection pressures, discriminating strategies dominate no matter how high the cooperate benefit is.

We have also performed similar calculations with another type of label-distributions, namely those arising from repeating short patterns. Similarly to the calculation above, one can calculate the relative stationary probability of finding the boundary at two points one period apart. The results generally don’t reduce to a form as simple as that above, but it is straight forward to plot the transition-line at which the relative probabilities equal 1, using numerical methods. These transition-lines can be found in the Supplement Section 3.2 for repeated patterns of length one to six. It is worth mentioning that not all distributions have a critical selection pressure, above which discriminating strategies will dominate independently of the cooperation benefit as, for example, the value found in the’single green label’-scenario. In the perfectly alternating case (green, blue, green, blue, …), for example, the transition line is completely independent of the selection pressure, and lies exactly at cooperation benefit of *b* = 2.

## Discussion

In this paper, we have introduced and studied a minimal model in which evolutionary dynamics can promote discriminating behaviour between arbitrary labels resulting in a reduction of the overall level of cooperation. Our model describes the cultural evolution of competing behaviours/strategies, of which some are discriminating, in the sense that they imply different behaviours towards agents carrying different labels. We have observed that the discriminating strategies end up dominating the system when the selection pressure is high — that is, when agents have a much higher tendency to imitate the behaviour of their most successful neighbour. The result is a hierarchical society of “winners” and “loosers” where agents carrying one label end up with a higher payoff than those carrying the other. The observed hierarchy is an example of spontaneous symmetry-breaking, as it emerges even though the model treats the labels symmetrically, and there is no extrinsic preference for choosing one strategy over any other. In fact, agents choose their strategy completely independently of their own label. As a consequence the low status agents (those with the lowest payoff) end up exhibiting out-group favouritism, by cooperating only with those neighbours having a different label than their own. This type of behaviour is in qualitative agreement with experimental data suggesting that members of certain low-status groups tend to express significantly lower in-group favouritism, or even favouring high-status group members^3–11^. These observations are usually explained with system justification theory^12^. System justification theory states that humans have an intrinsic drive for justifying – and thereby validating – established structures around them. Compared to the well-established psychological theory of system justification, our model is a minimalistic toy-model. We have ignored many important aspects of human nature, as well as making unrealistic assumptions, in an attempt to make the model as simple as possible, while still qualitatively capturing the observed phenomenon. Our model is intended as an abstract investigation of the idea, that mechanisms for hierarchical discrimination could be initiated or reinforced via group-dynamical mechanisms outside of individual preferences. We do not, however, understand this as a replacement for the established theory, but rather as a complementary mechanism.

It is worth noting that our model is fundamentally different from the established theory of tag-based cooperation^17–23^. In models of tag-based cooperation, tags are passed on to future generation together with the strategy of the parent, unless a rare mutation occurs. A successful combination of tag and strategy will therefore quickly invade the system, resulting in a population where a large majority of agents have the same tag. In the model presented in this paper, agents never change their label, and agents with one label can adopt the strategy of neighbours with the other label. Consequently the dynamics differ dramatically in two ways.

Firstly, we find a strong tendency for our system to settle into a stationary macro-distribution of strategies. This is in sharp contrast to the oscillatory (or wave-like) behaviour of previous models. In these models, the system is almost always susceptible to invasion from new strategies. When the population is dominated by complete defection, it is vulnerable to the random emergence of an agent with a currently non-existing tag and an in-group favouring strategy. Later, when that agent-type has taken over most of the population, it becomes susceptible to free-riders who have the same tag, but do not cooperate with their peers.

Secondly, when discriminating strategies become dominant in models of tag-based cooperation, they do so by the fortune of a strong correlation between a given tag and an in-group favouring strategy. When we observe discriminating strategies dominating in our model, it tends to form large (mesoscale) regions each dominated by a single strategy—either “cooperate only with green” or “cooperate only with blue”. Within each of these regions, the population is generally a mix of agents with different labels. Consequently a non-negligible fraction of the population is actually expressing out-group favouritism.

In our model, strategies are able to produce the richest individuals by letting the agents carrying one label exploit those carrying the other. This helps explaining the observed connection between a high selection pressure, *w*, and the dominance of discriminating strategies. The selection pressure can be interpreted as the agent’s ‘eagerness’ to copy their richest neighbour. When it is strong enough, the contagion benefit of strategies that produce richer agents outperforms the disadvantage of also producing poor ones. If we keep this analysis in mind we can also argue, that a strategy with explicit in-group favouritism (“cooperate only with neighbours carrying the same label as myself”) would not have an evolutionary advantage under these dynamics. With such a strategy no agents would have the advantage of receiving donations from their neighbours without giving back.

The models of discrimination which have followed a classical economical approach by assuming rational agents^14,15^ also cannot capture the cause of discrimination that we have observed in our model. This is because it is crucial to the development of hierarchical discrimination in our model that agents do not act intelligently in the direction of self-interest. Even in economical models where agents have imperfect information about each other, they are typically assumed to at least have full information about the fundamental rules of the game they are playing. It can be argued that evolutionary game theory makes the general assumption that agents have no understanding about cause and effect in their social interactions, and thus must rely on observed correlations between other agents’ behaviour and their profit, in order to try to optimize their game. Such an assumption seems more likely in the context of unconscious behaviours, or in complex social interactions where the causal connection between actions and delayed rewards is in fact indirect.

Many interesting questions remain in the context of the non-group-based mechanism of discrimination in social systems that we studied in a minimal model in this paper. In particular, we have enforced a spatial structure on the model-population which is one out of a number of well known ways to promote cooperation in prisoner’s dilemma type games^16,24^. At this point it remains an open question whether a cooperation-reducing, hierarchical discrimination will also emerge via spontaneous symmetry-breaking when introducing meaningless tags into models where cooperation is achieved by different means.

### Accession codes

All code used to produce the included figures has been submitted as Supplementary Material.

## Supplementary information

supplementary material

figure_generation
