# Supplementary material for: Imitating the winner leads to discrimination in spatial prisoner’s dilemma model
Source: Sci Rep. 2019 Mar 7;9:3776. doi: 10.1038/s41598-019-40583-w (PMC6405999; doi:10.1038/s41598-019-40583-w)

Supplementary material to

Imitating the winner leads to discrimination in spatial prisoner's dilemma model

Gorm Gruner Jensen* **and Stefan Bornholdt**

Institute for Theoretical Physics, University of Bremen, Bremen, 28359, Germany
*ggjensen@itp.uni-bremen.de

1 Phase diagrams for variations of graphs and label-distributions

All panels in the following three figures display parameter scans similar to that in figure 2 in the main text, but showing different macroscopic observables. Each of the four right-most panels in each figure show the fraction of agents with a given strategy. The captions describe the strategy behaviour towards blue and green respectively, e.g. (coop/defect) mean cooperate with blue neighbours only. *'label/strat-correlation'* measures to what extent agents positively discriminate their own label. Add one for every agent who has a cooperating strategy towards their own badge, and subtract one for each agent cooperating with the label they don't carry themselves, and normalize by the number of agents.

1.1 Uniform label distribution

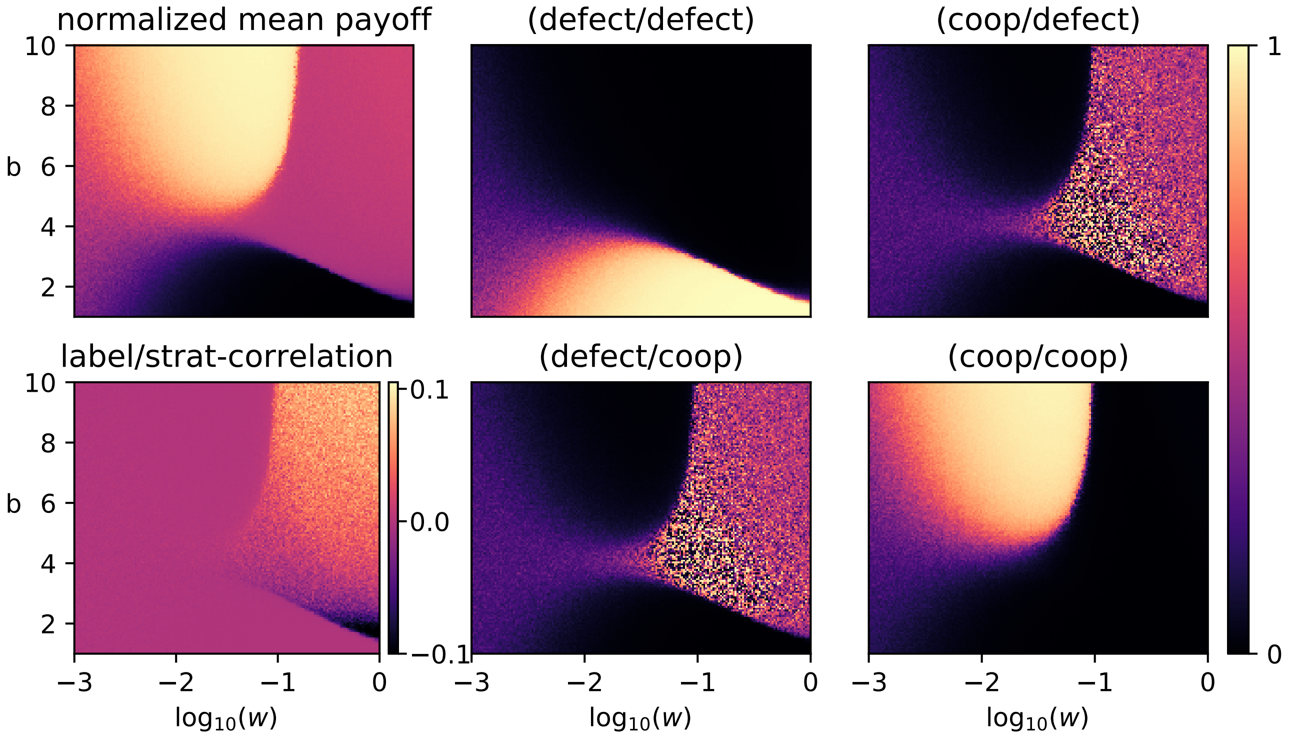

The parameter-scan shown here differs from the model described in the main text only by having a uniform label distribution. Every agent has 50/50 percent chance of being blue or green. The label distribution is random and redrawn from a uniform distribution at every data point.

1.2 Random Graphs - average connectivity 4

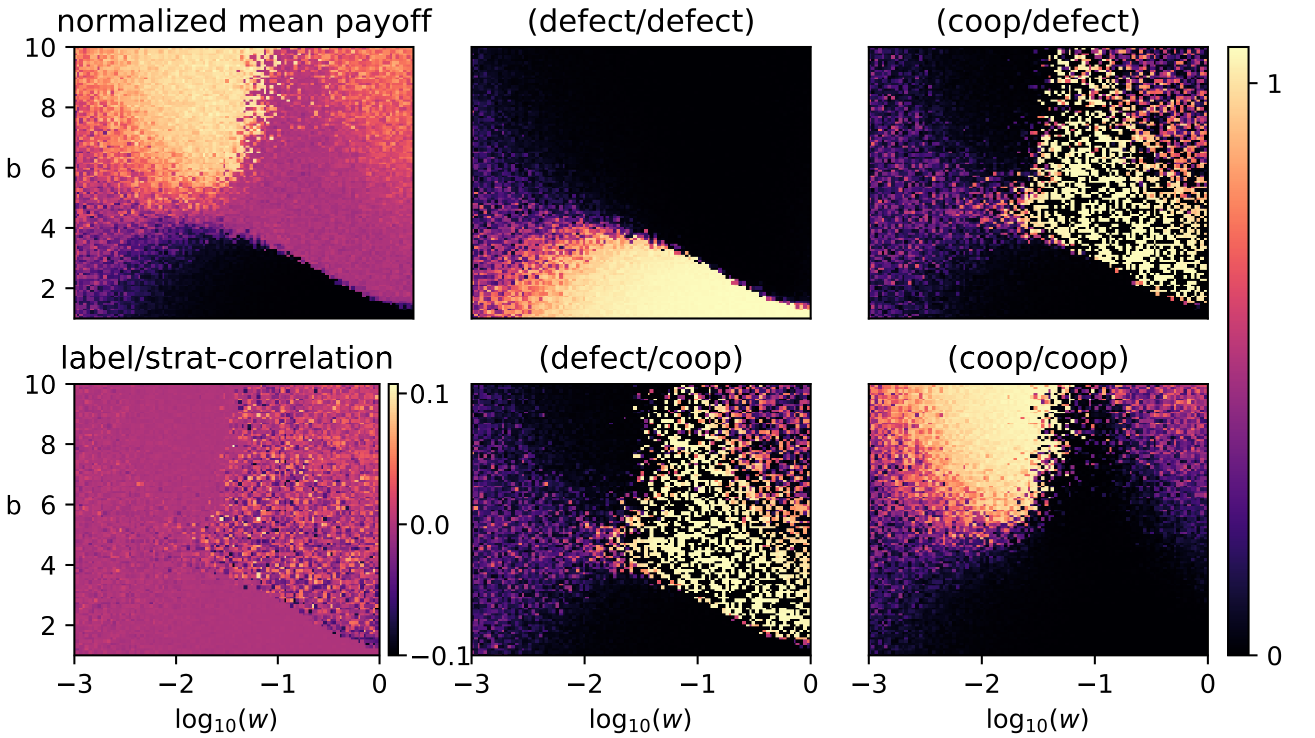

As the model described in this paper is defined in terms of local interactions, it is straightforward to extent the investigations to arbitrary graph topologies. The parameter-scan shown here is executed on binomial random-graphs with 1000 nodes and average connectivity 4. A new random graph, and a new random uniform label-distribution is drawn for every time series.

1.3 1D - Ring

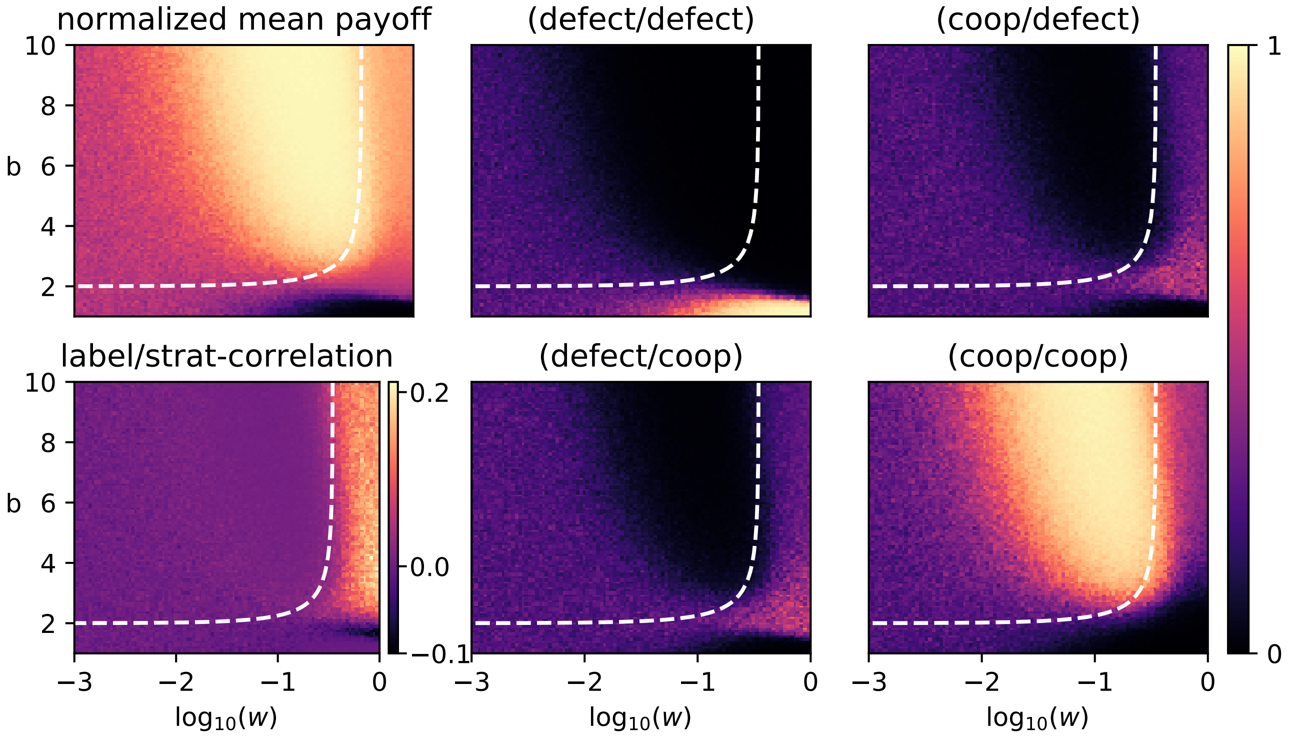

For comparison with the theoretically predicted phase-transition mentioned in the paper (red line), we here show what the phase diagram looks like for a 1D system, i.e. a line of agents with two neighbours each, closed at the ends to form a ring. The label distribution is random and redrawn from a uniform distribution at every data point.

2 Timeseries - Converging to stable population fractions

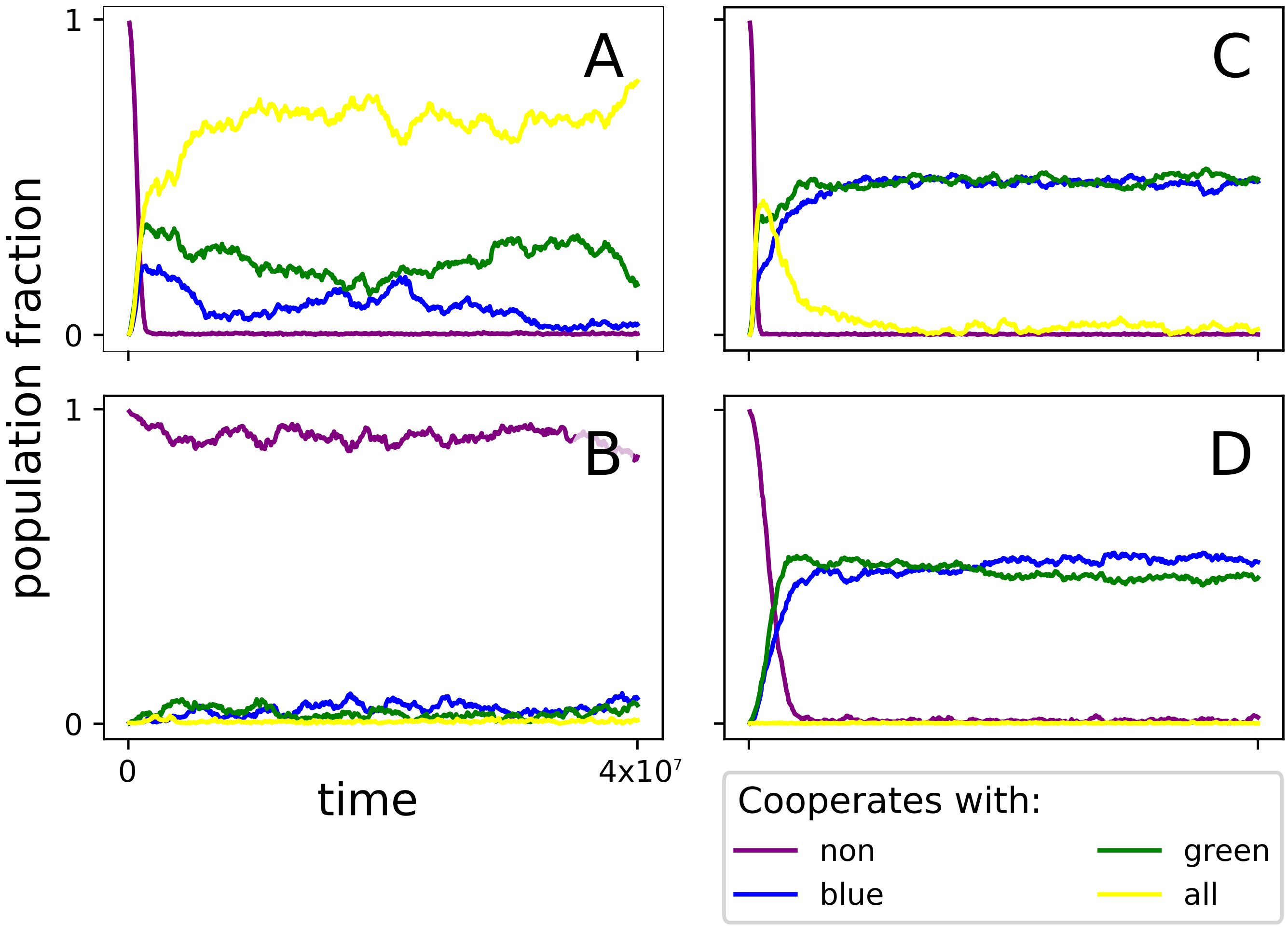

These four panels show the time-series of the population fraction of each of the four strategies, corresponding to each of the snap-shots in figure 2 in the in the main text. Each simulation starts with a population of 100% defectors.

The figures strongly suggest, that the model dynamics reaches a stationary state, with some stochastic fluctuations around stationary strategy-distributions.

3 Analytic solution of 1D system with a single green agent

3.1 A single green agent

As mentioned in main text, the phase-transition can be analytically calculated in a simplified version of the 1D system with out mutation ($\mu = 0$).

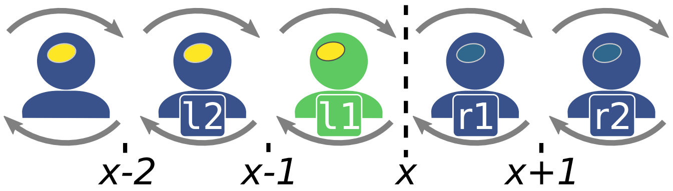

Imagine a line of agents, each connected to their two nearest neighbours. One agent in the middle of the line has the green label, while all others have the blue label. The dynamical variable in this system is a point on the line, x, such that all agents to the left of that point have the yellow strategy (cooperate with everyone), and all agents to the right have the blue strategy (cooperate only with blue neighbours). At any point in time, we enumerate the agents according to how far they are from the border. The first agent to the right (left) is called $r1$ ($l1$), the second $r2$ ($l2$), and so on.

Since the mutation is assumed to be zero, the border between the two strategies can only move if the agent immediately at one side copies the strategy of the agent on the other.

The boundary moves left if the first agent left of the boundary ($l1$) imitates the strategy of the first player right of the boundary ($r1$). This happens with probability:

$$P(x \to x-1) = \frac{1}{N}\frac{f_{r1}}{f_{r1}+f_{l2}} = \frac{1}{N}\frac{1}{1+f_{l2}/f_{r1}} = \frac{1}{N}\frac{1}{1+\exp\left(w\cdot(p_{l2}-p_{r1})\right)},$$

given the boundary is at position x, where f_i and p_i are the fitness and payoff of agent i, and $l2$ is the second agent left of the boundary. Likewise the probability that the boundary will move right is:

$$P(x \to x+1|x) = \frac{1}{N}\frac{1}{1+\exp\left(w\cdot(p_{r2}-p_{l1})\right)},$$

where $r2$ is the second agent left of the boundary. After having determined the individual stepping probabilities we can calculate the stationary distribution of the boundary by assuming microscopic balance:

$$P(x)P(x \to x+1|x) = P(x)P(x+1 \to x|x+1),$$

where P(x) is the stationary probability that the boundary is at position x. From this we get an expression for the fraction between the stationary probability of a position x and at a position $x+n$:

$$\frac{P(x)}{P(x+n)} = \frac{P(x+1 \to x|x+1)}{P(x \to x+1|x)} \cdot \frac{P(x+2 \to x+1|x+2)}{P(x+1 \to x+2|x+1)} \cdot \ldots \cdot \frac{P(x+n \to x+n-1|x+n)}{P(x+n-1 \to x+n|x+n-1)}.$$

If we assume x is a position at least three steps to the completely cooperative side of the green agent (the discriminating strategy dominates) and $x+n$ is a position at least three steps to the discriminating side (the completely cooperative strategy dominates) then we obtain the following expression after reducing factors which appear in both numerator and denominator.

$$\frac{P(\text{discrimination dominates})}{P(\text{full cooperation dominates})} = \frac{1+e^{-2wb}}{2}\frac{(1+e^{w})^2}{(1+e^{-w})^2} = \frac{e^{2w}}{\tanh wb+1}$$

When the fraction is greater than one, we expect a strategy of asymmetric cooperation (i.e. discrimination) to be able to outperform the full cooperation in a mixed population. Comparison with the phase diagram for the one-dimensional system show a close, but non-perfect match (see supplementary figure 1.3). In the limit of very high cooperation benefit, $b \to \infty$, this condition reduces to:

$$w > \log 2/2$$

This result shows, that no matter how high the cooperation-benefit is, there will always be a critical selection-pressure above which discriminating strategies are more likely to spread than unconditional cooperation.

3.2 1D system with periodic label-distributions

In a one-dimensional system with a label-distribution generated by repeating a simple pattern, one can calculate the relative probability of finding the boundary between two strategies at two points one period apart. The figures below show the transition-line between dominance of "cooperate with all" and "cooperate only with blue", i.e. the line at which the relative probability between these two strategies is equal to one. Each figure is labelled with a representation of the repeated label-pattern. x stand for "green", i.e. the label which is only cooperated with by agents left of the boundary point, and o stand for "blue", i.e. the label that all agents are always cooperating with.

The calculations follow almost exactly the same procedure as described for the label-distribution with one single green label. For each possible position of the boundary between the two strategies, one has to calculate both the probability that the boundary will move left, and the probability that it will move right. The relative probability of finding the boundary at two points one "cycle" apart can then be found by using the exact same equation as before.

Cycle length 1:

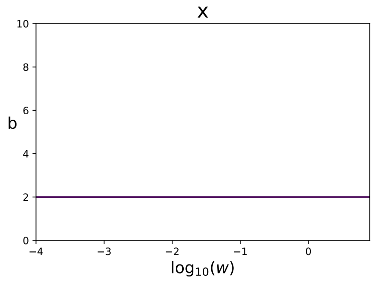

Cycle length 2:

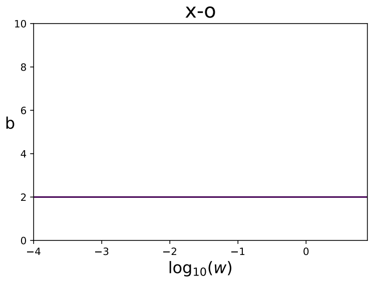

Cycle length 3:

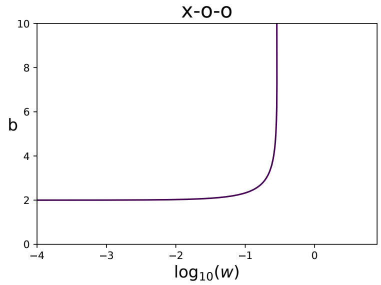

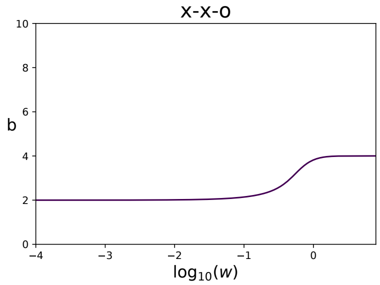

Cycle length 4:

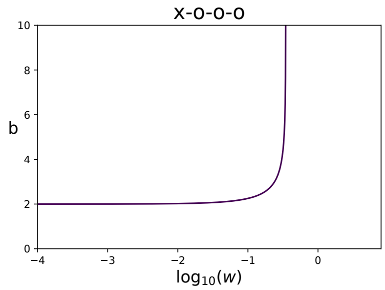

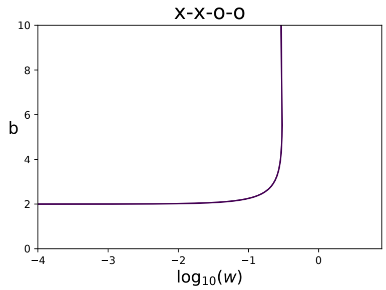

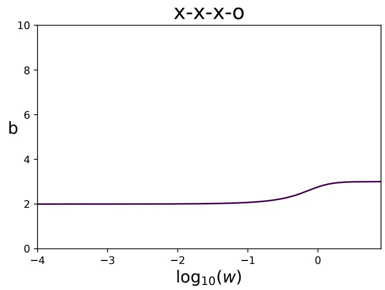

Cycle length 5:

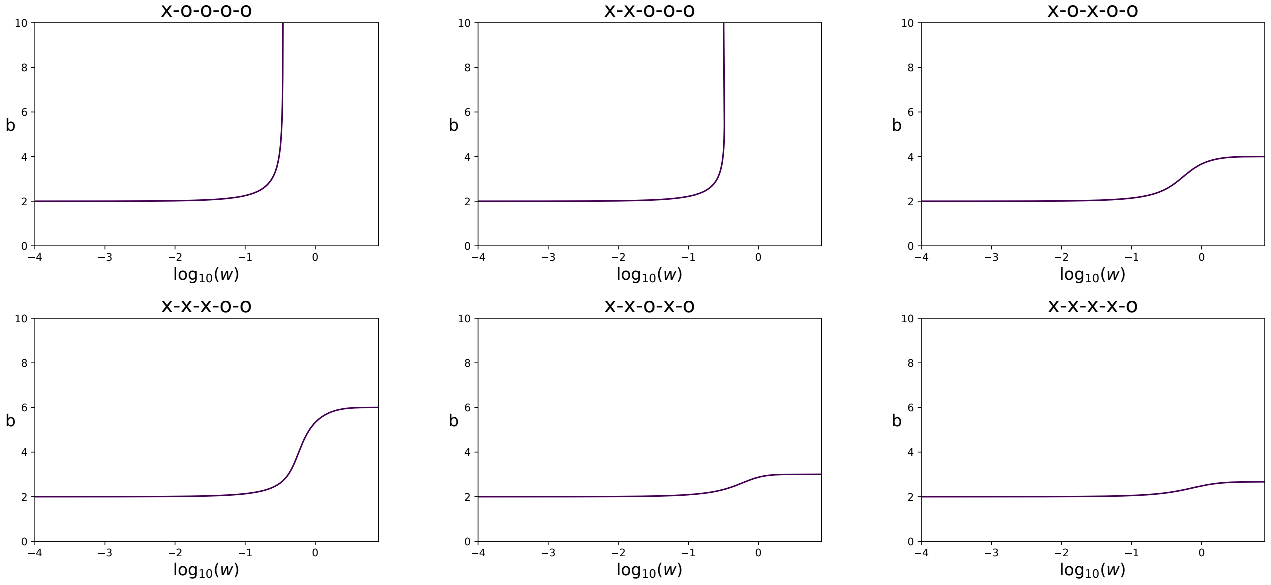

Cycle length 6:

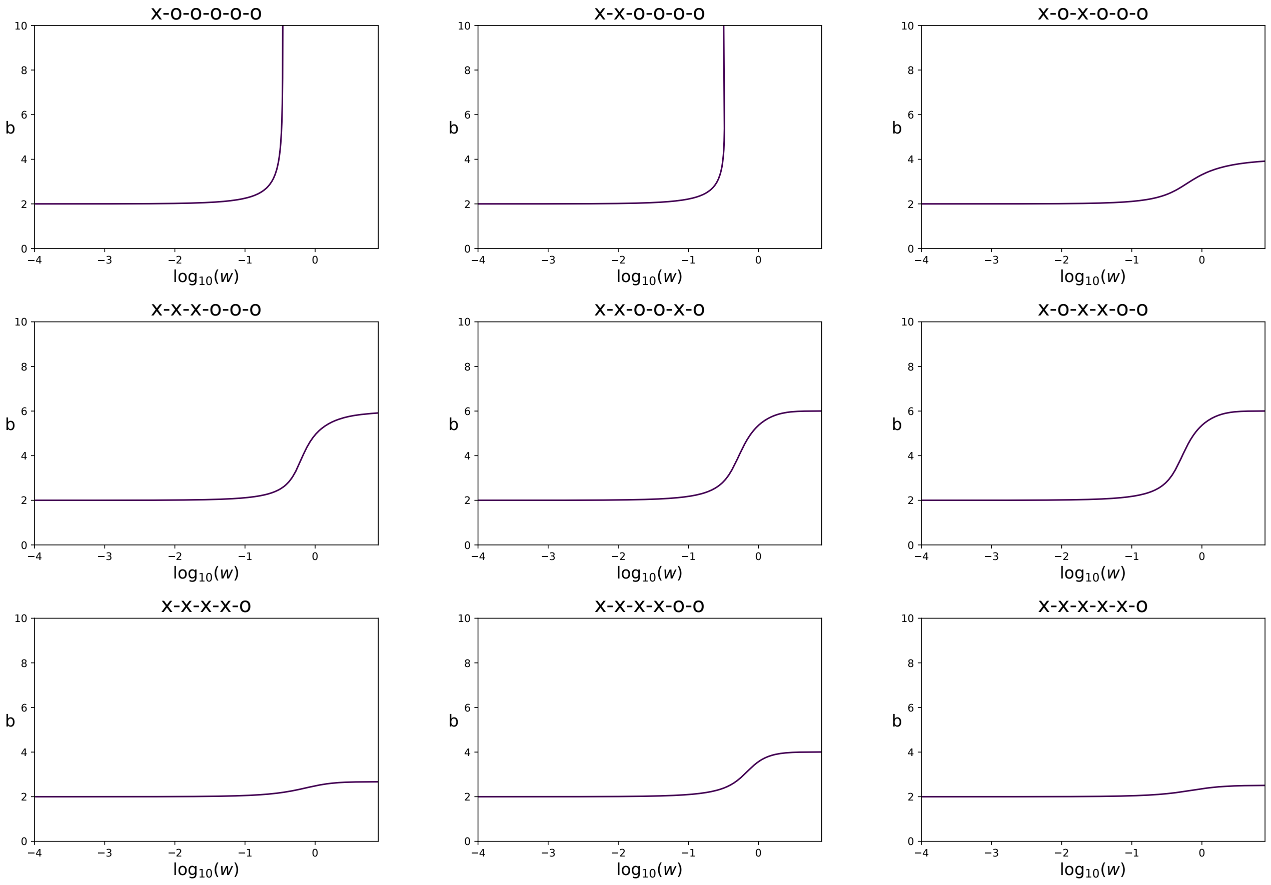

Supplement: Supplementary file 1 — supplementary material [file 41598_2019_40583_MOESM1_ESM.pdf]
