# Supplementary material for: Imitating the winner leads to discrimination in spatial prisoner’s dilemma model
Source: Sci Rep. 2019 Mar 7;9:3776. doi: 10.1038/s41598-019-40583-w (PMC6405999; doi:10.1038/s41598-019-40583-w)
Supplement: Supplementary file 2 — figure_generation [file 41598_2019_40583_MOESM2_ESM.zip › figure_generation/randomGraphScan/wbScan_randGraph.pdf]

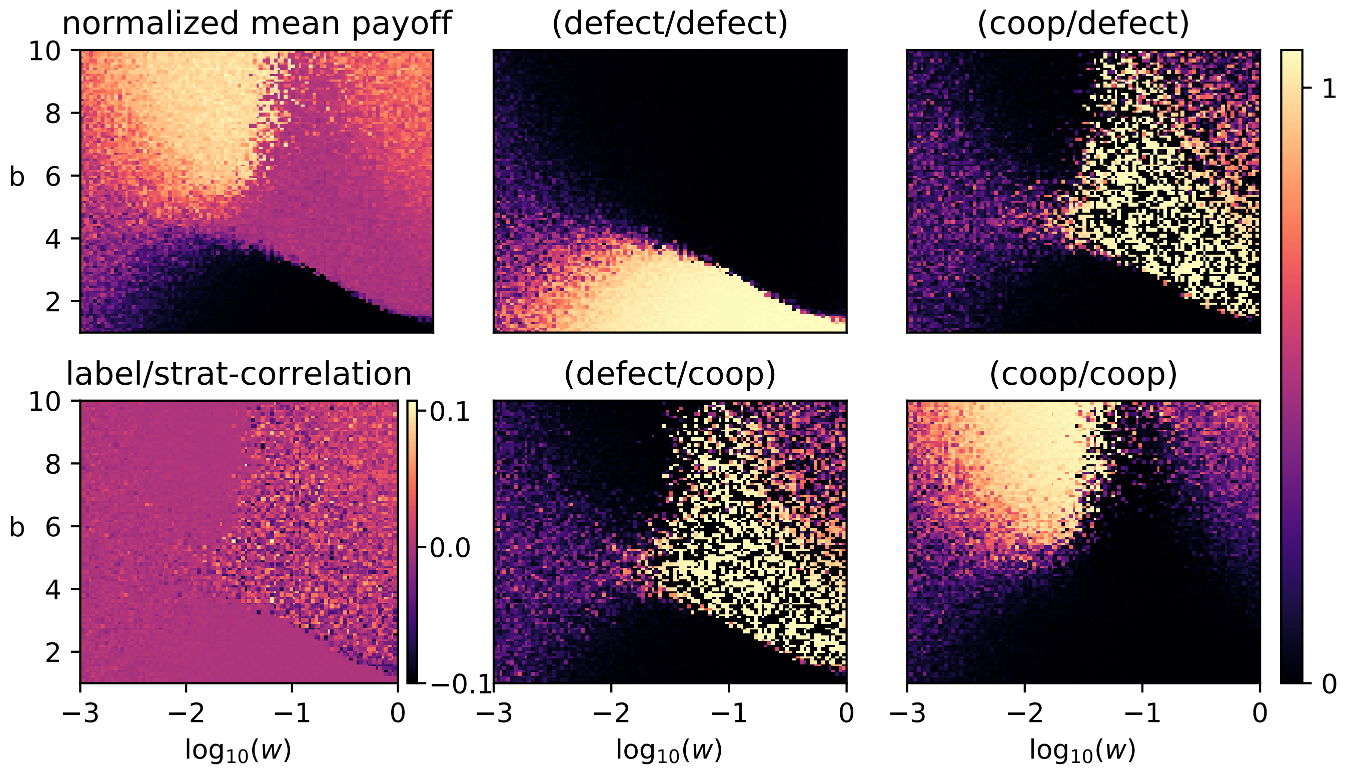

normalized mean payoff
(defect/defect)
(coop/defect)
label/strat-correlation
(defect/coop)
(coop/coop)
b
10
8
6
4
2
0.1
0.0
−0.1
1
0
−3
−2
−1
0
log10(w)
