# Supplementary figures and images for: Imitating the winner leads to discrimination in spatial prisoner’s dilemma model
Source: Sci Rep. 2019 Mar 7;9:3776. doi: 10.1038/s41598-019-40583-w (PMC6405999; doi:10.1038/s41598-019-40583-w)

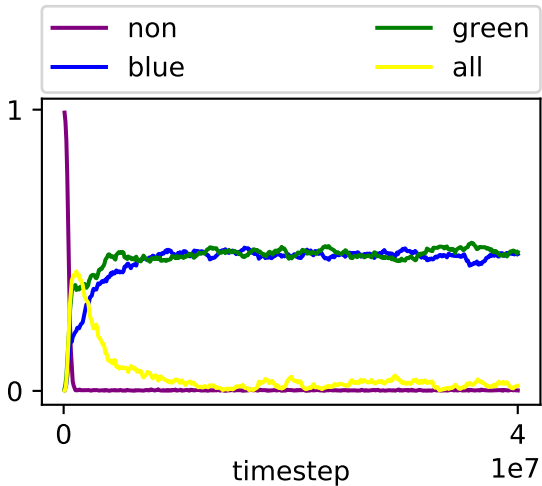

Supplement: Supplementary file 2 — figure_generation [file 41598_2019_40583_MOESM2_ESM.zip › figure_generation/single_run/timeseries.pdf]

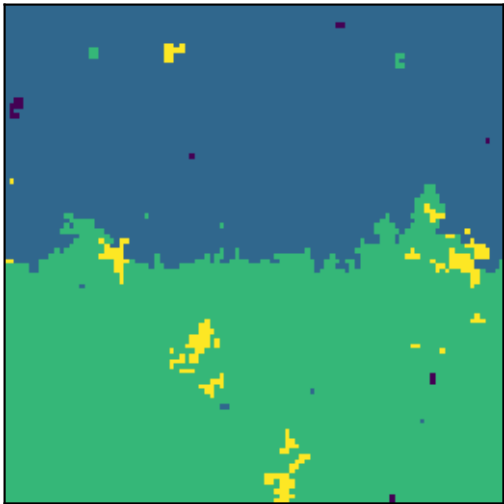

Supplement: Supplementary file 2 — figure_generation [file 41598_2019_40583_MOESM2_ESM.zip › figure_generation/single_run/endDist.pdf]

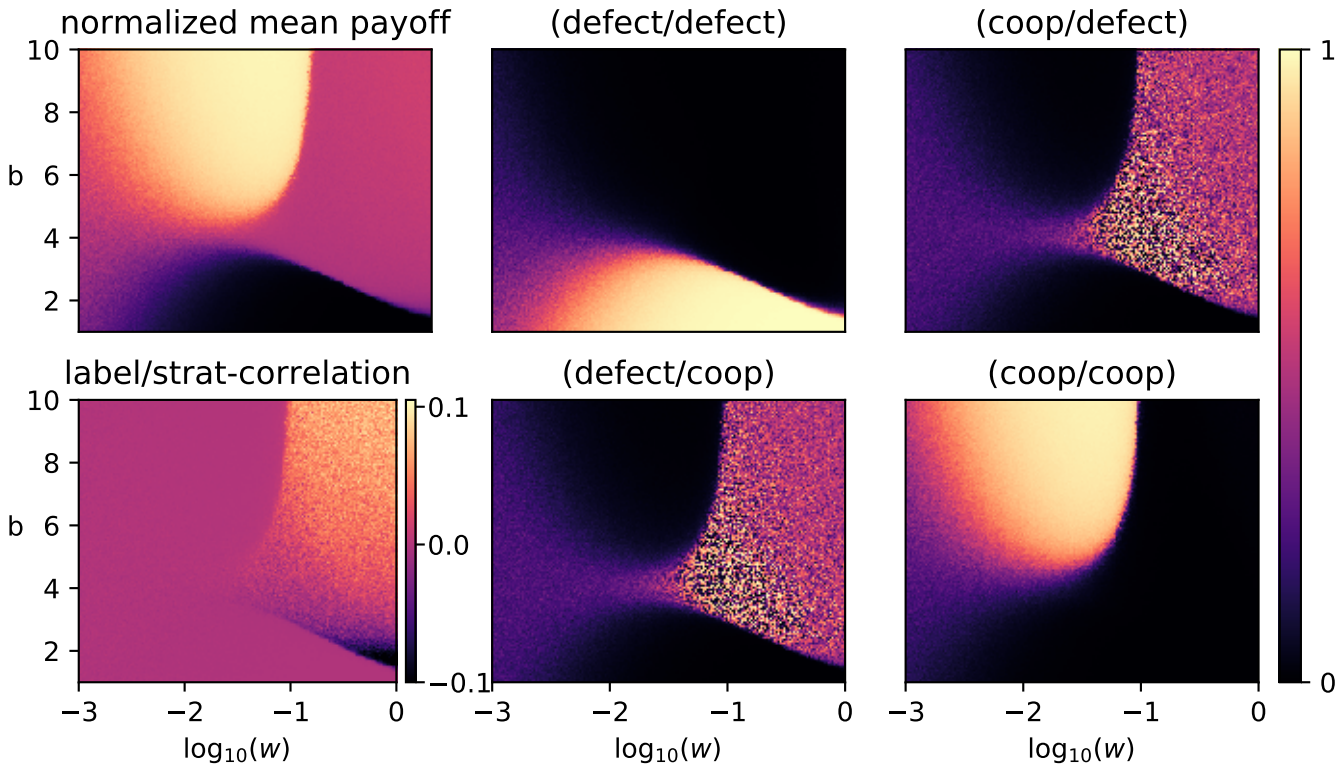

Supplement: Supplementary file 2 — figure_generation [file 41598_2019_40583_MOESM2_ESM.zip › figure_generation/uniformLabelDistScan/uniform-label-scan.pdf]

Cycle length 1:

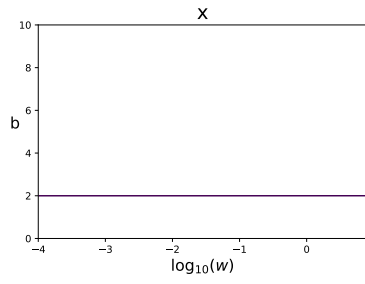

Cycle length 2:

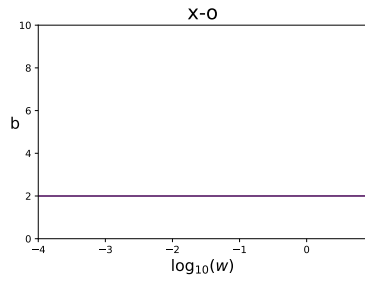

Cycle length 3:

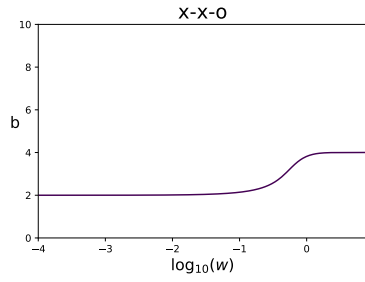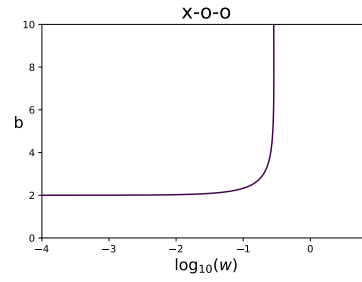

Cycle length 4:

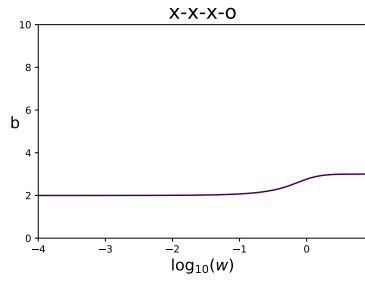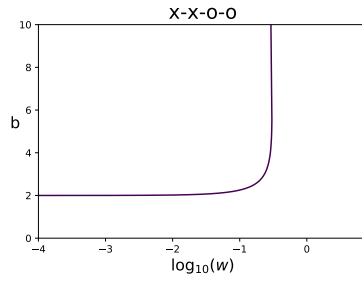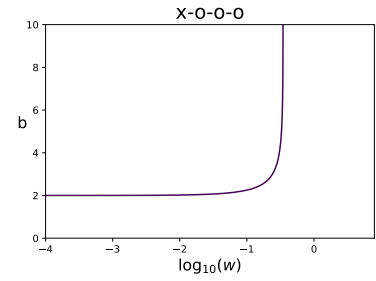

Cycle length 5:

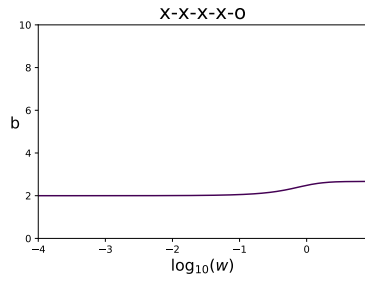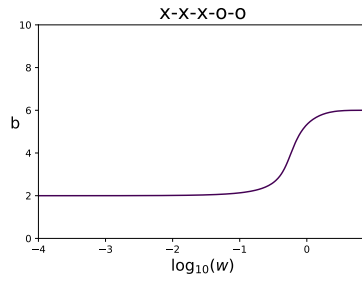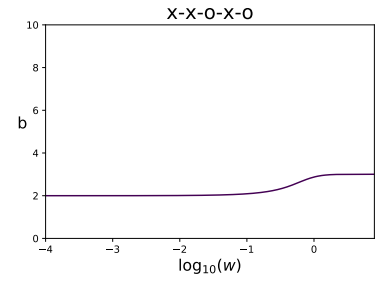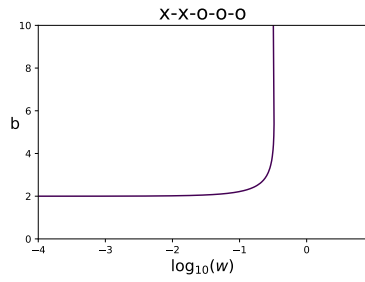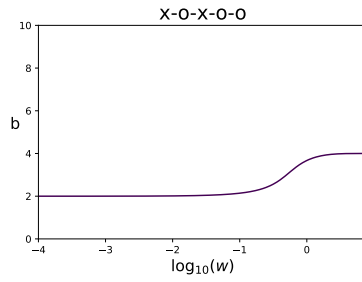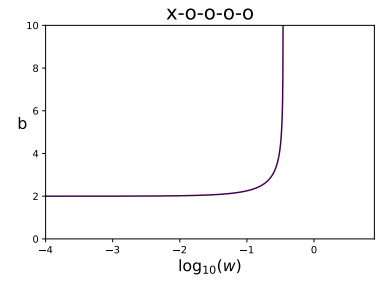

Cycle length 6:

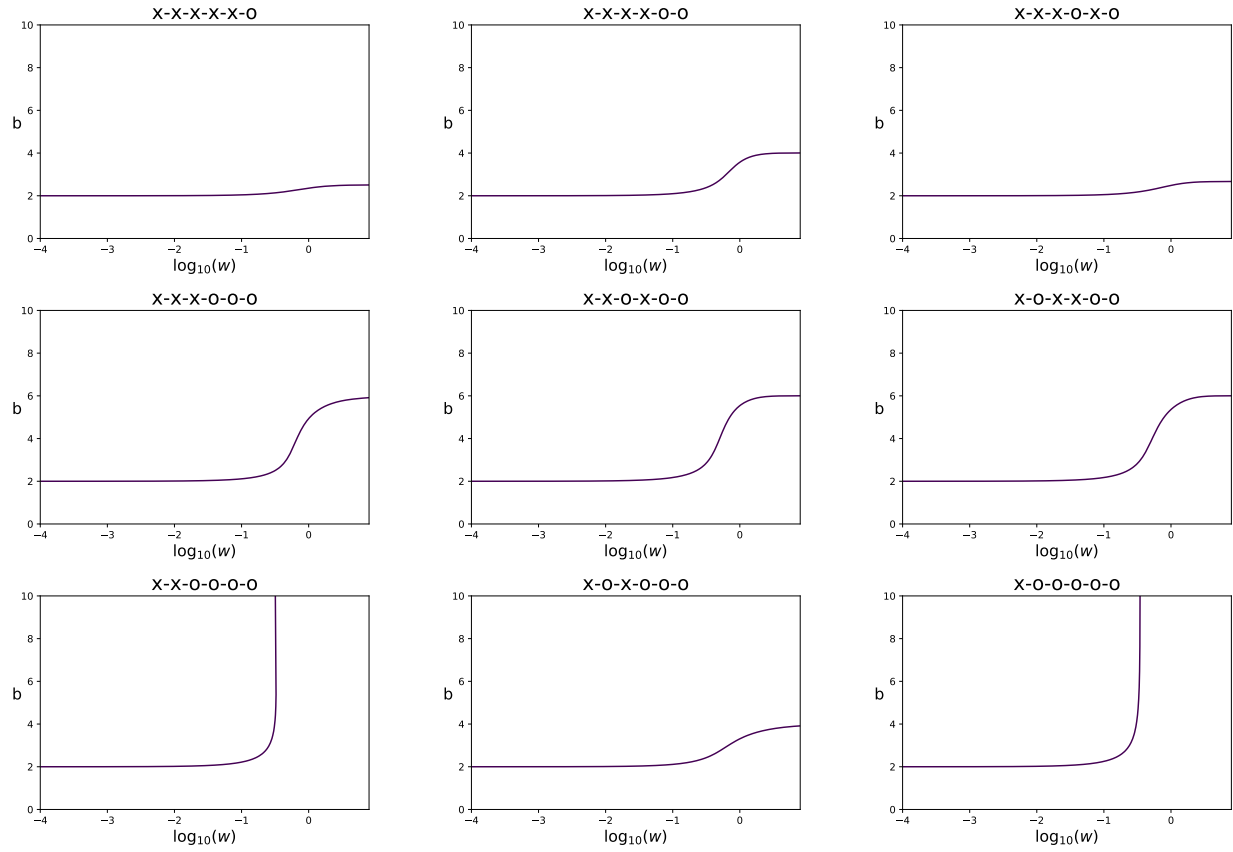

Supplement: Supplementary file 2 — figure_generation [file 41598_2019_40583_MOESM2_ESM.zip › figure_generation/1d_cyclic_labels/collection.pdf]

X

b

10

8

6

4

2

0

-4

-3

-2

-1

0

$\log_{10}(w)$

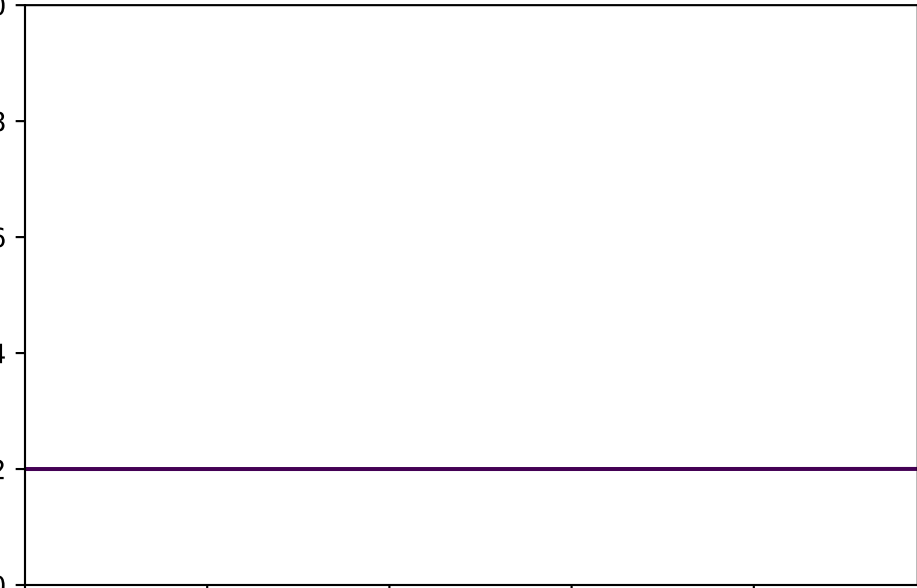

Supplement: Supplementary file 2 — figure_generation [file 41598_2019_40583_MOESM2_ESM.zip › figure_generation/1d_cyclic_labels/x.pdf]

X-O

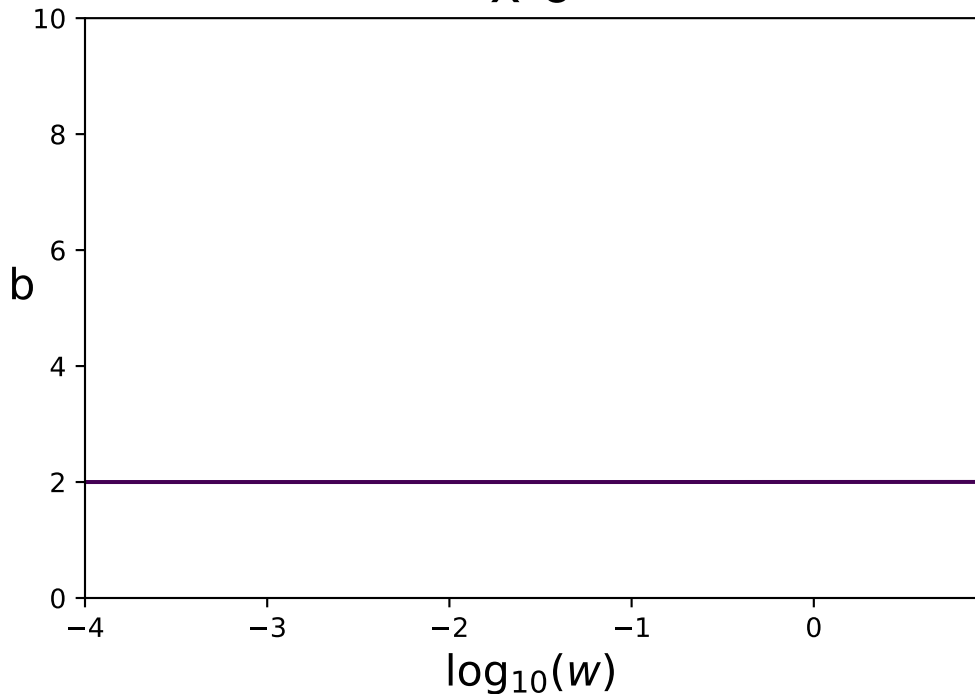

Supplement: Supplementary file 2 — figure_generation [file 41598_2019_40583_MOESM2_ESM.zip › figure_generation/1d_cyclic_labels/xo.pdf]

X-X-O

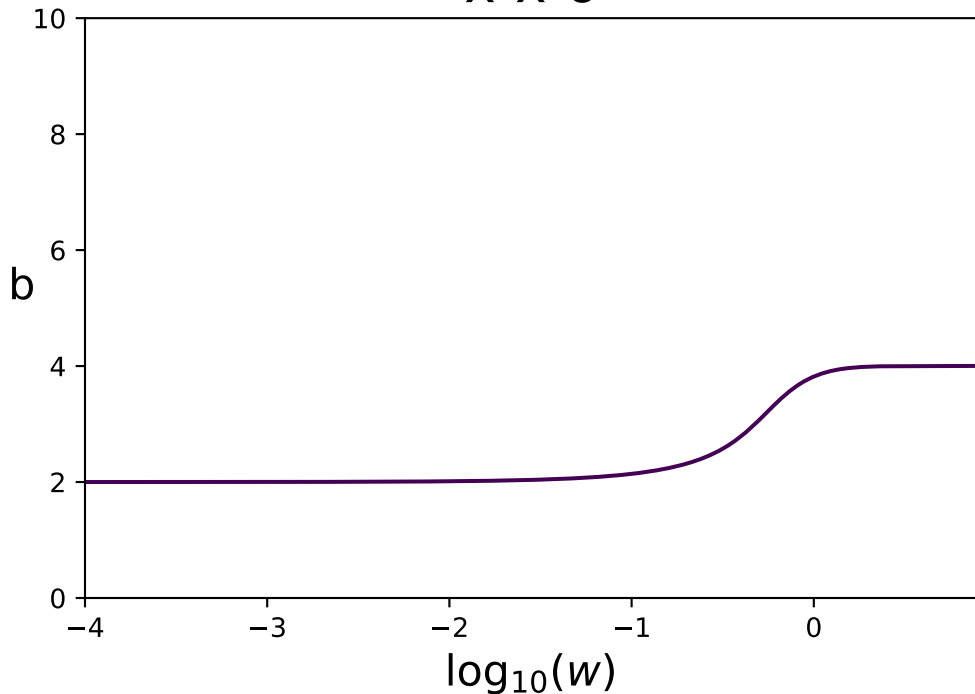

Supplement: Supplementary file 2 — figure_generation [file 41598_2019_40583_MOESM2_ESM.zip › figure_generation/1d_cyclic_labels/xxo.pdf]

X-O-O

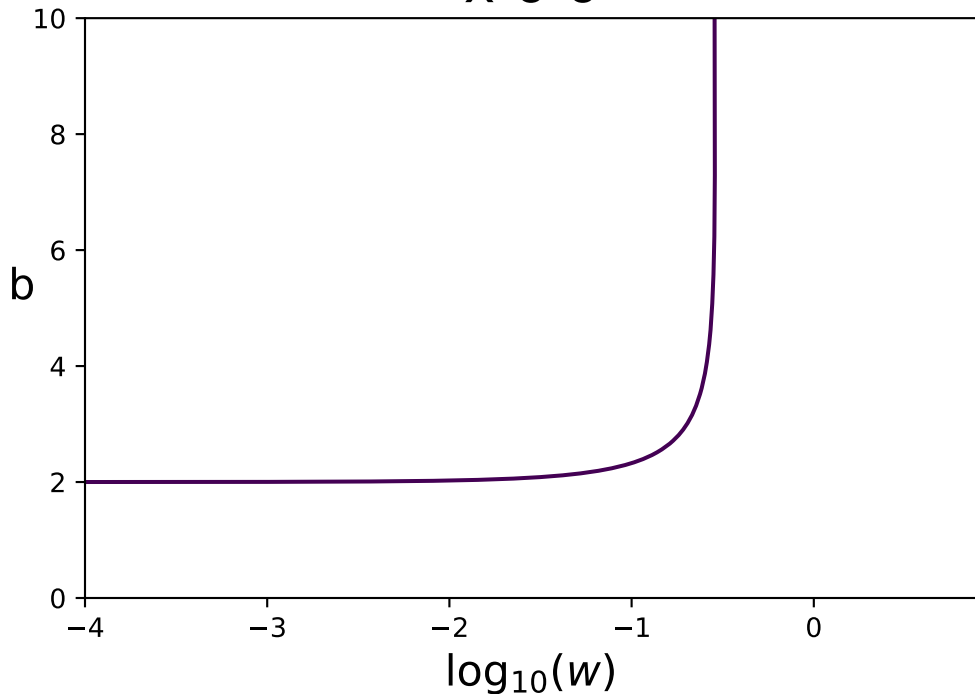

Supplement: Supplementary file 2 — figure_generation [file 41598_2019_40583_MOESM2_ESM.zip › figure_generation/1d_cyclic_labels/xoo.pdf]

X-X-X-O

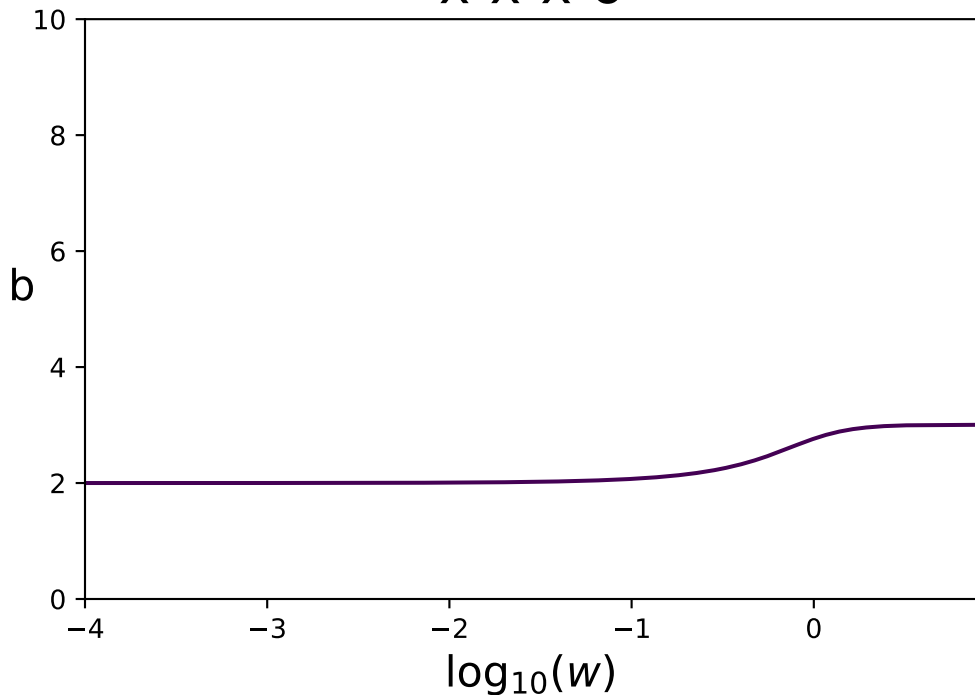

Supplement: Supplementary file 2 — figure_generation [file 41598_2019_40583_MOESM2_ESM.zip › figure_generation/1d_cyclic_labels/xxxo.pdf]

X-X-O-O

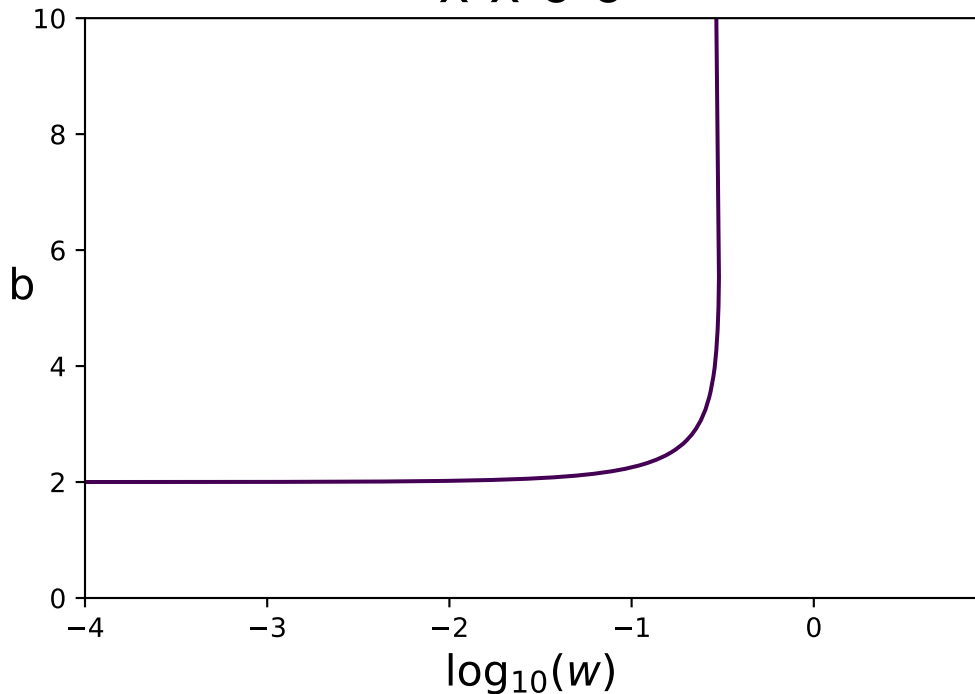

Supplement: Supplementary file 2 — figure_generation [file 41598_2019_40583_MOESM2_ESM.zip › figure_generation/1d_cyclic_labels/xxoo.pdf]

X-O-O-O

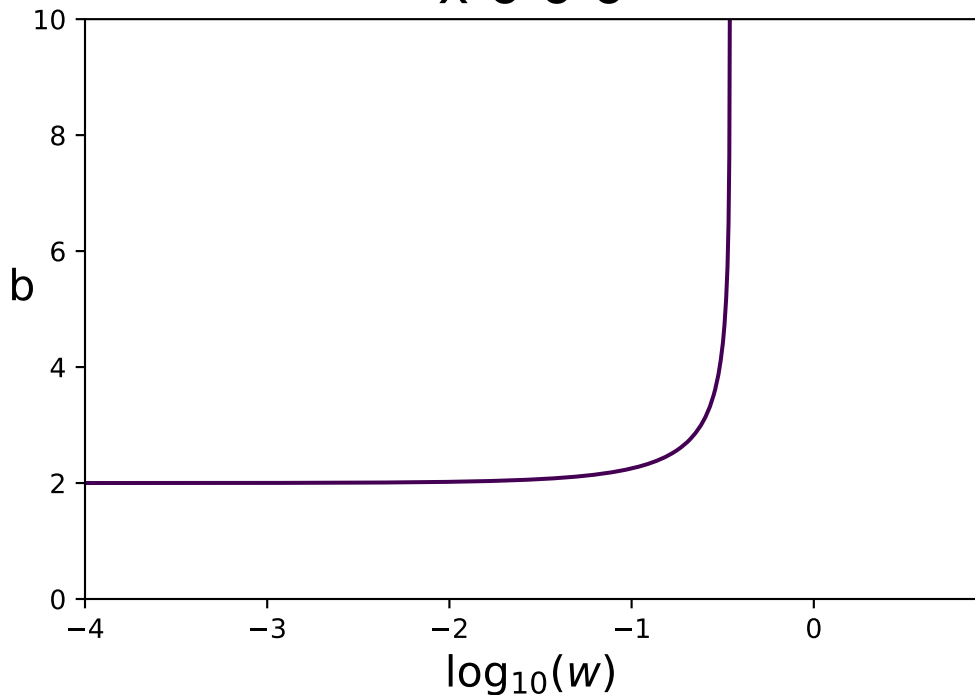

Supplement: Supplementary file 2 — figure_generation [file 41598_2019_40583_MOESM2_ESM.zip › figure_generation/1d_cyclic_labels/xooo.pdf]

X-0-0-0-0

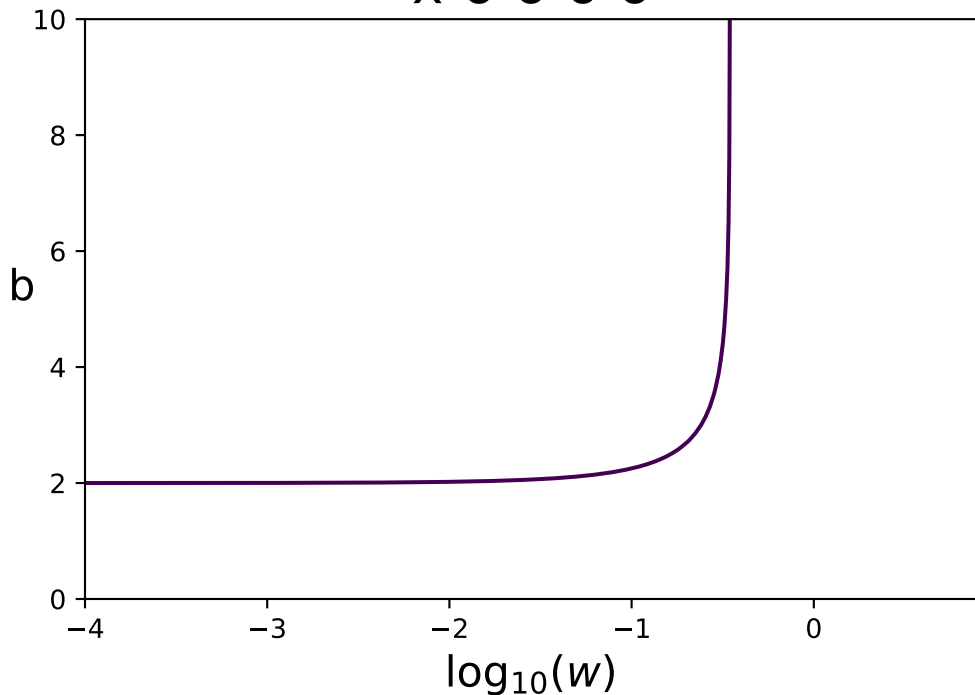

Supplement: Supplementary file 2 — figure_generation [file 41598_2019_40583_MOESM2_ESM.zip › figure_generation/1d_cyclic_labels/xoooo.pdf]

X-X-O-O-O

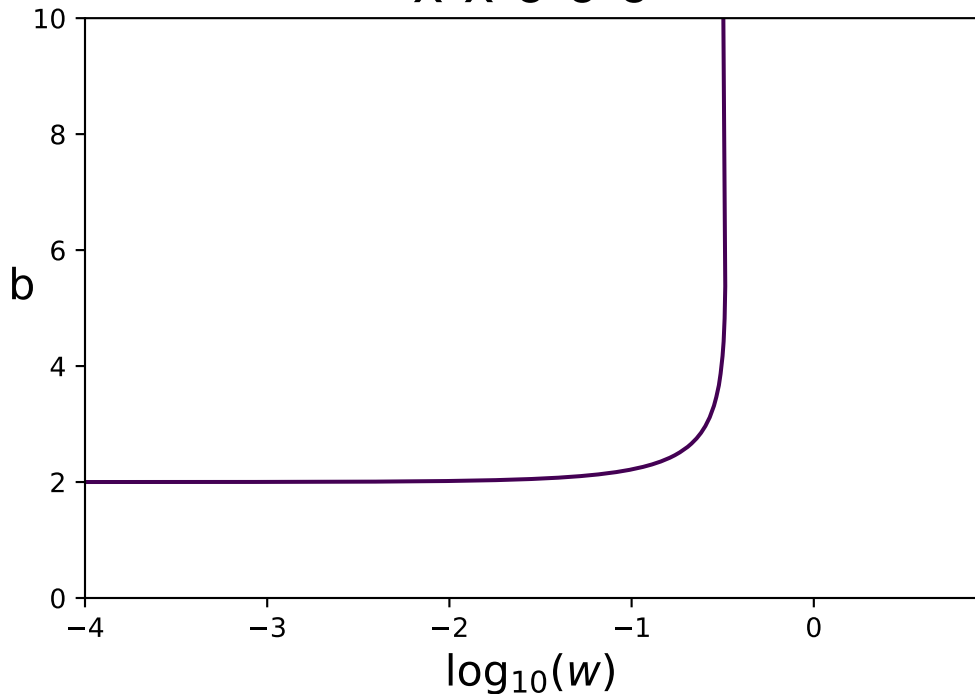

Supplement: Supplementary file 2 — figure_generation [file 41598_2019_40583_MOESM2_ESM.zip › figure_generation/1d_cyclic_labels/xxooo.pdf]

X-O-X-O-O

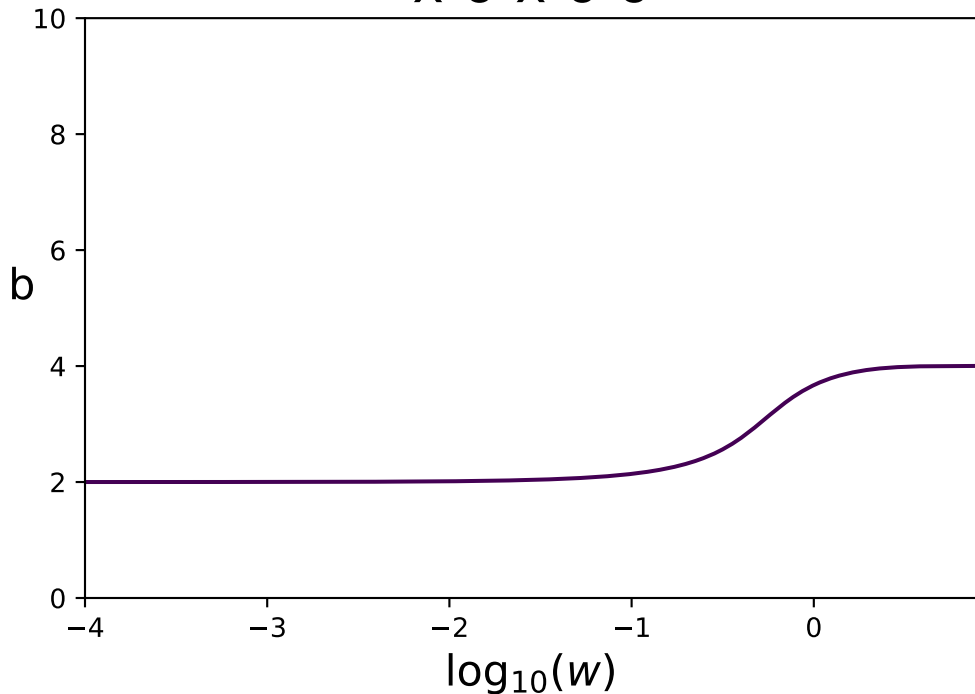

Supplement: Supplementary file 2 — figure_generation [file 41598_2019_40583_MOESM2_ESM.zip › figure_generation/1d_cyclic_labels/xoxoo.pdf]

X-X-O-X-O

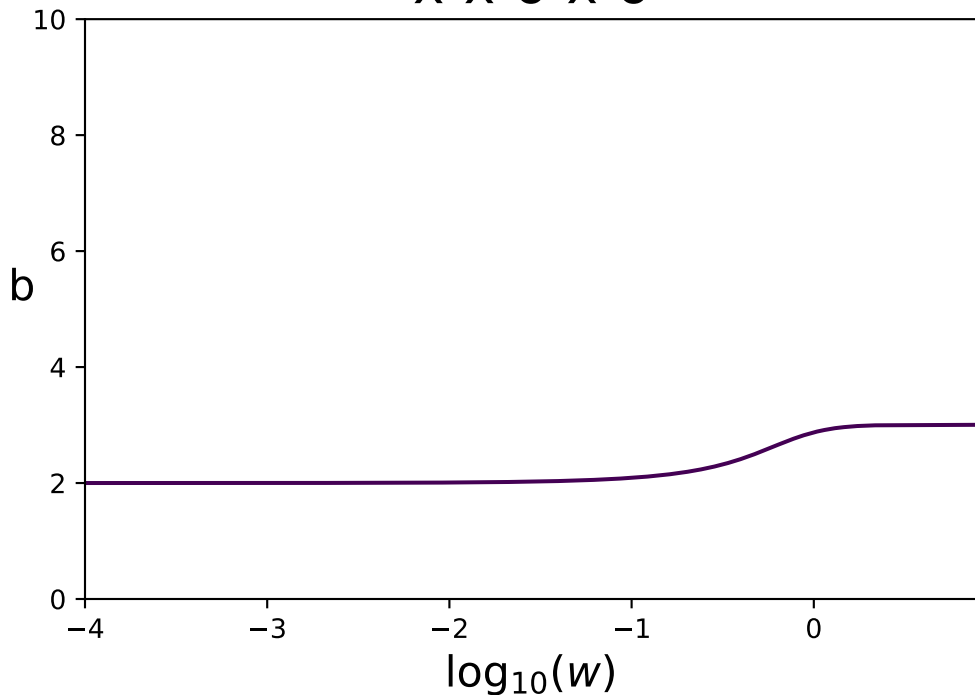

Supplement: Supplementary file 2 — figure_generation [file 41598_2019_40583_MOESM2_ESM.zip › figure_generation/1d_cyclic_labels/xxoxo.pdf]

X-X-X-O-O

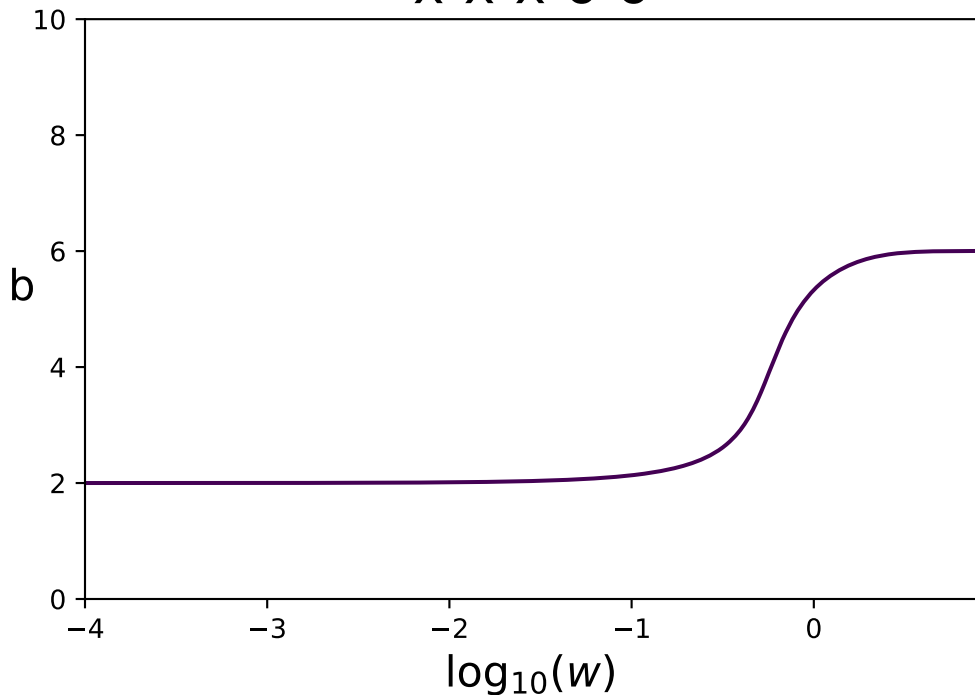

Supplement: Supplementary file 2 — figure_generation [file 41598_2019_40583_MOESM2_ESM.zip › figure_generation/1d_cyclic_labels/xxxoo.pdf]

X-X-X-X-O

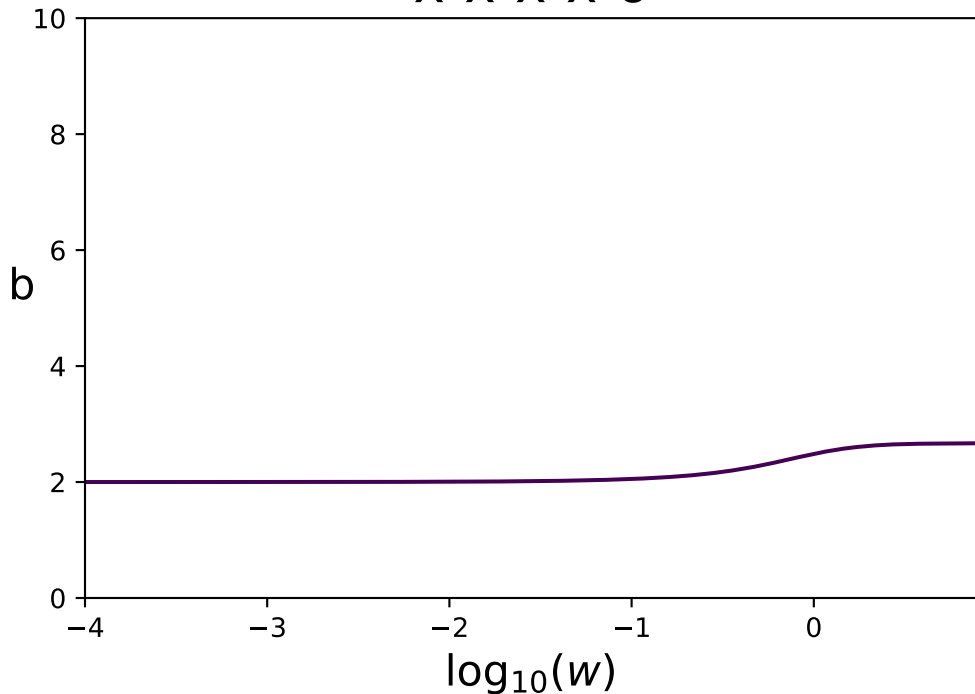

Supplement: Supplementary file 2 — figure_generation [file 41598_2019_40583_MOESM2_ESM.zip › figure_generation/1d_cyclic_labels/xxxxo.pdf]

X-X-X-X-X-O

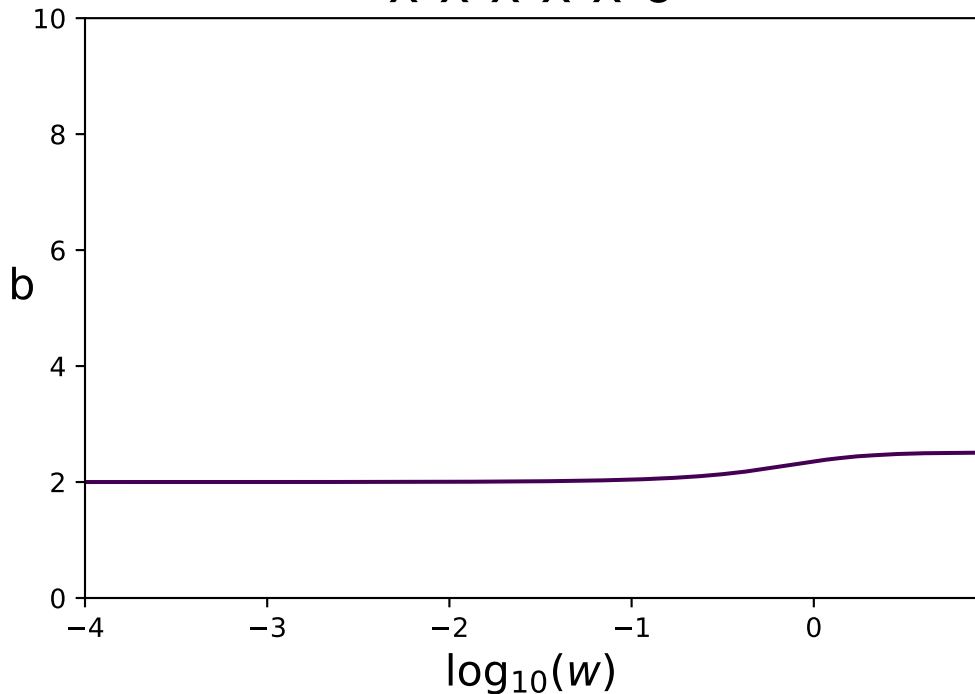

Supplement: Supplementary file 2 — figure_generation [file 41598_2019_40583_MOESM2_ESM.zip › figure_generation/1d_cyclic_labels/x-x-x-x-x-o.pdf]

X-X-X-X-O-O

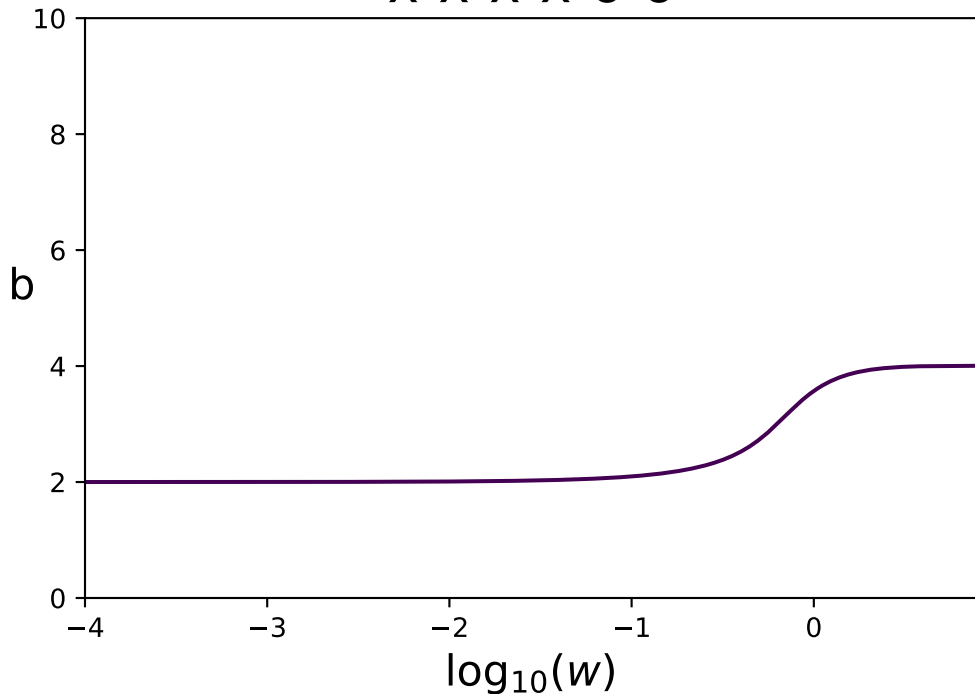

Supplement: Supplementary file 2 — figure_generation [file 41598_2019_40583_MOESM2_ESM.zip › figure_generation/1d_cyclic_labels/x-x-x-x-o-o.pdf]

X-X-X-O-X-O

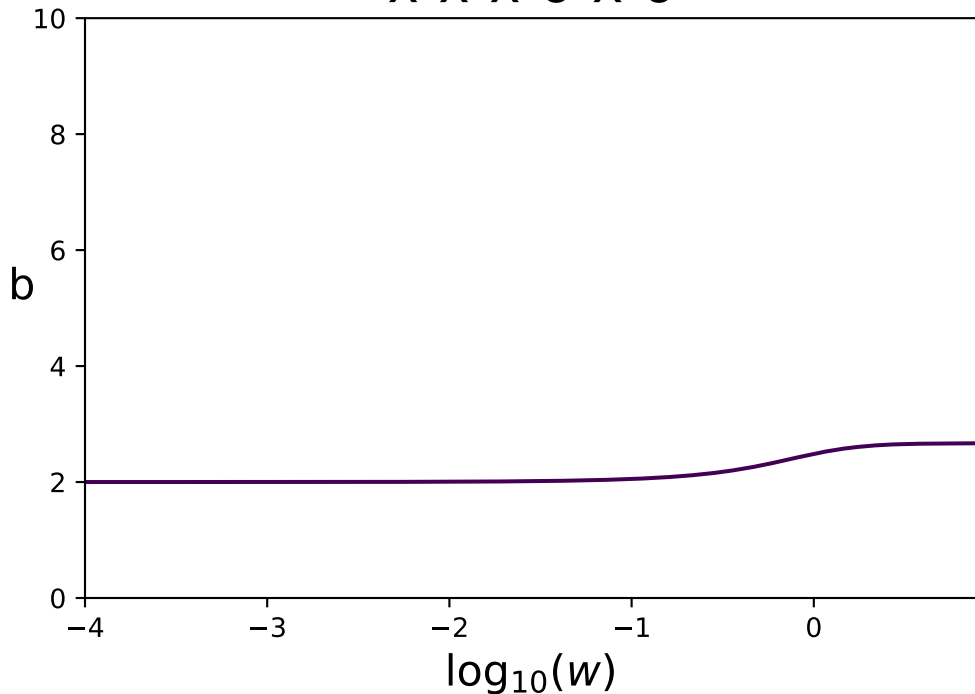

Supplement: Supplementary file 2 — figure_generation [file 41598_2019_40583_MOESM2_ESM.zip › figure_generation/1d_cyclic_labels/x-x-x-o-x-o.pdf]

X-X-X-O-O-O

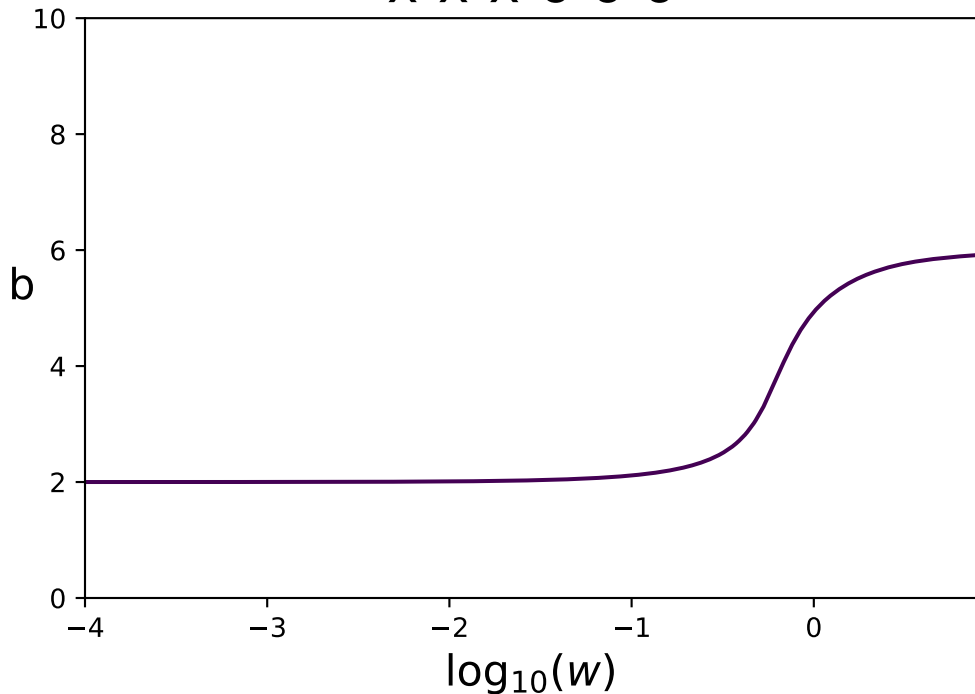

Supplement: Supplementary file 2 — figure_generation [file 41598_2019_40583_MOESM2_ESM.zip › figure_generation/1d_cyclic_labels/x-x-x-o-o-o.pdf]

X-X-O-X-O-O

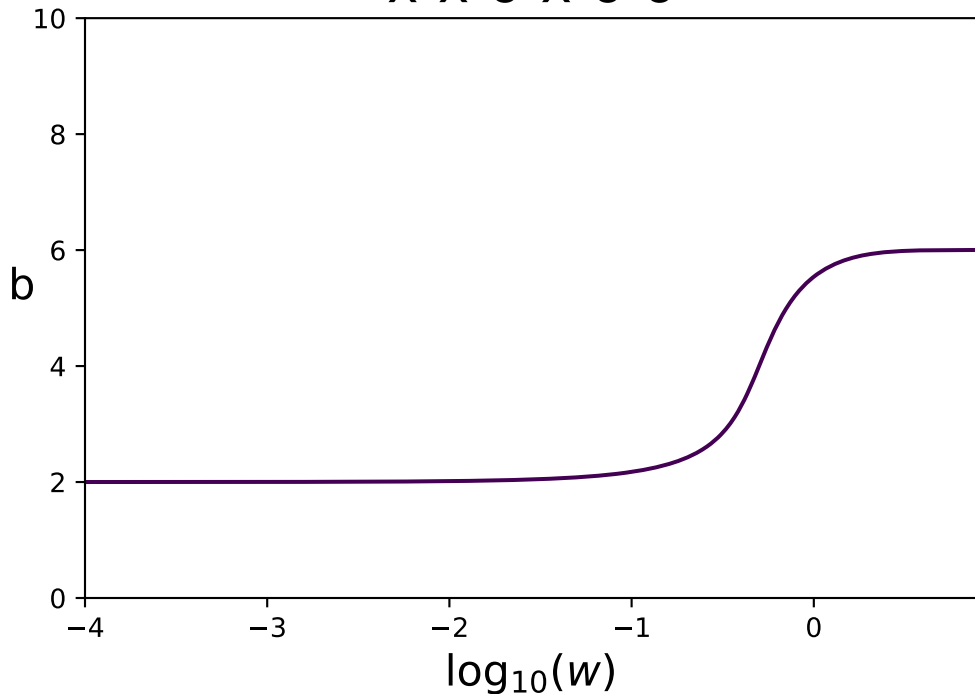

Supplement: Supplementary file 2 — figure_generation [file 41598_2019_40583_MOESM2_ESM.zip › figure_generation/1d_cyclic_labels/x-x-o-x-o-o.pdf]

X-X-O-O-X-O

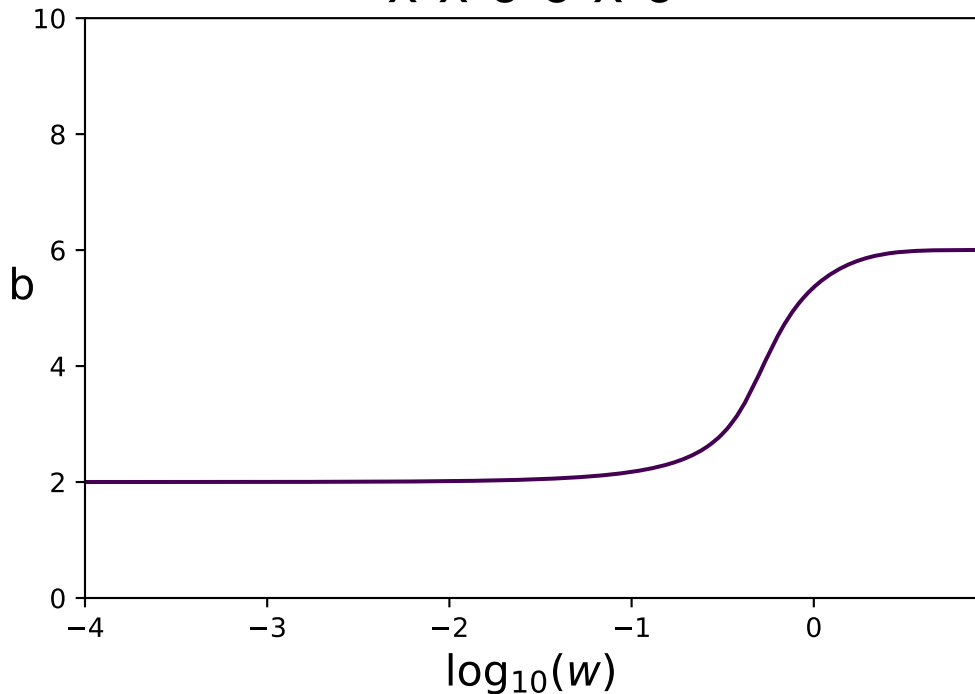

Supplement: Supplementary file 2 — figure_generation [file 41598_2019_40583_MOESM2_ESM.zip › figure_generation/1d_cyclic_labels/x-x-o-o-x-o.pdf]

X-O-X-X-O-O

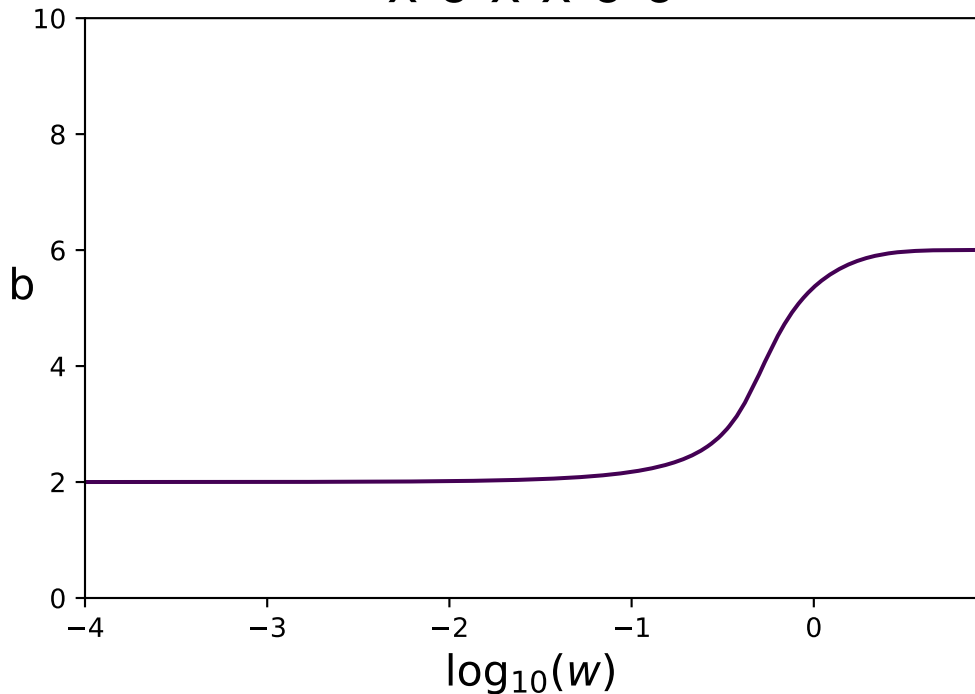

Supplement: Supplementary file 2 — figure_generation [file 41598_2019_40583_MOESM2_ESM.zip › figure_generation/1d_cyclic_labels/x-o-x-x-o-o.pdf]

X-X-O-O-O-O

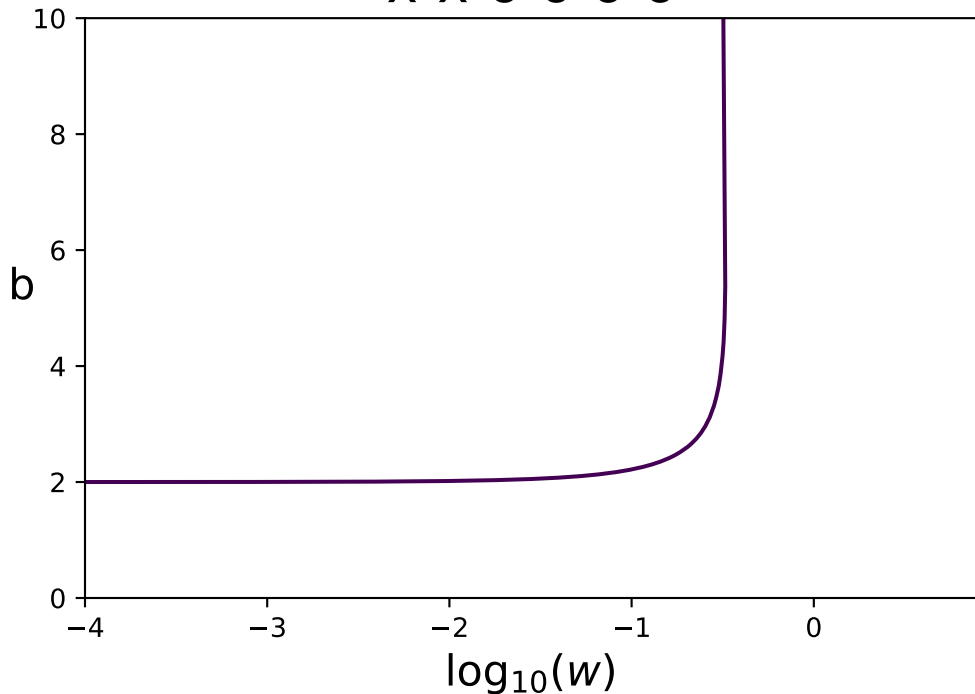

Supplement: Supplementary file 2 — figure_generation [file 41598_2019_40583_MOESM2_ESM.zip › figure_generation/1d_cyclic_labels/x-x-o-o-o-o.pdf]

X-O-X-O-O-O

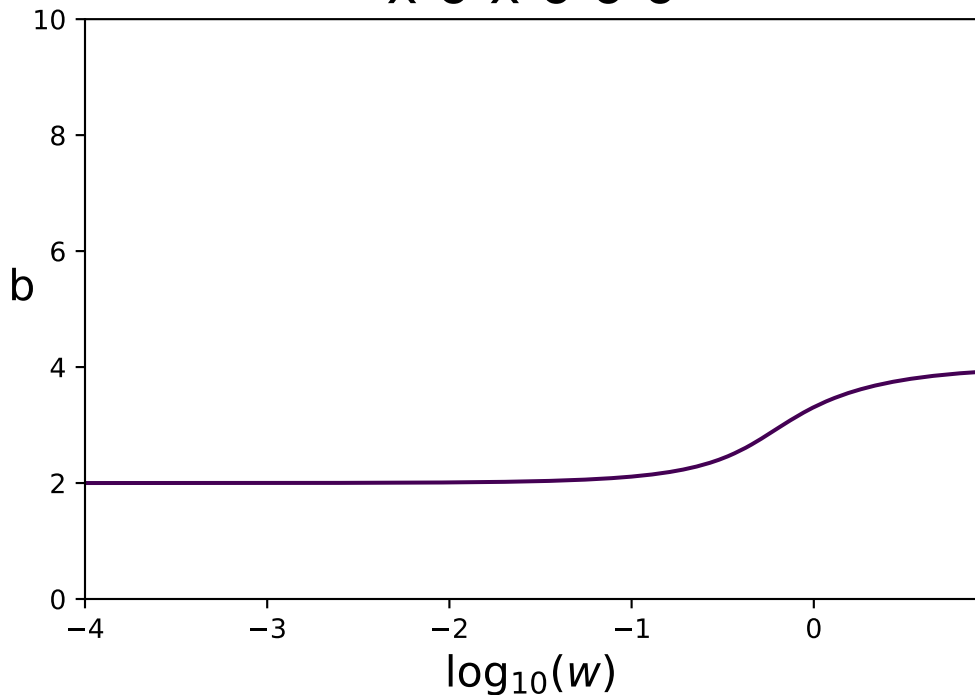

Supplement: Supplementary file 2 — figure_generation [file 41598_2019_40583_MOESM2_ESM.zip › figure_generation/1d_cyclic_labels/x-o-x-o-o-o.pdf]

X-O-O-O-O-O

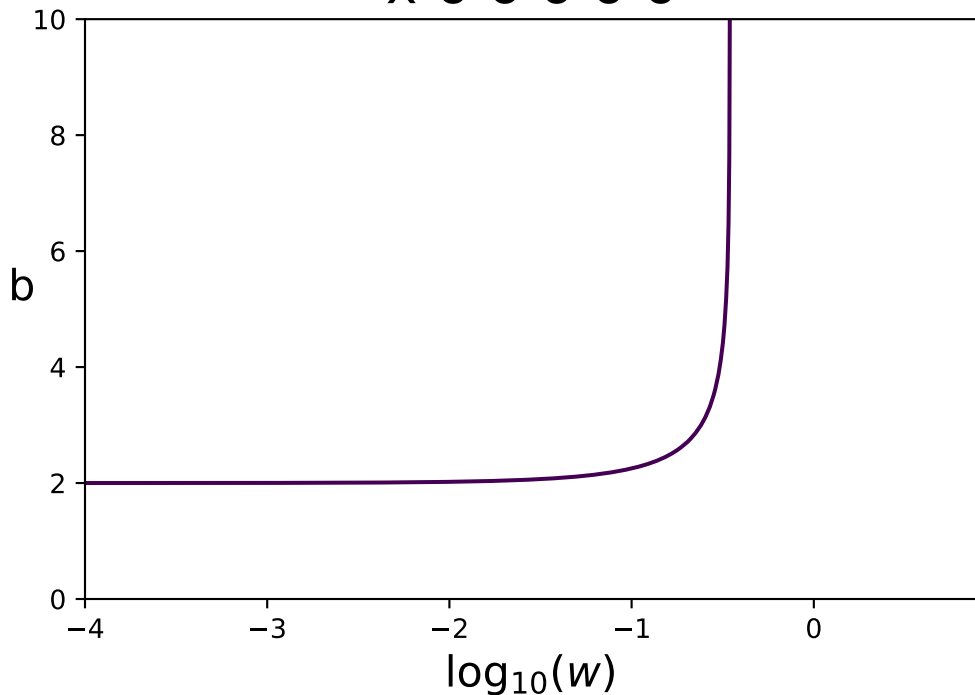

Supplement: Supplementary file 2 — figure_generation [file 41598_2019_40583_MOESM2_ESM.zip › figure_generation/1d_cyclic_labels/x-o-o-o-o-o.pdf]
